# Genetic Bypass of *Aspergillus nidulans crzA* Function in Calcium Homeostasis

**DOI:** 10.1534/g3.113.005983

**Published:** 2013-07-01

**Authors:** Ricardo S. Almeida, Omar Loss, Ana Cristina Colabardini, Neil Andrew Brown, Elaine Bignell, Marcela Savoldi, Sergio Pantano, Maria Helena S. Goldman, Herbert N. Arst, Gustavo H. Goldman

**Affiliations:** * Faculdade de Ciências Farmacêuticas de Ribeirão Preto, Brazil 14040-903; ‡ Section of Microbiology, Imperial College London, London SW7 2AZ, United Kingdom; § Biomolecular Simulations Group, Institut Pasteur de Montevideo, Uruguay 11400; ** Faculdade de Filosofia, Ciências e Letras de Ribeirão Preto, Universidade de São Paulo, São Paulo, Brazil 14040-901; †† Laboratório Nacional de Ciência e Tecnologia do Bioetanol–CTBE, Caixa Postal 6170, 13083-970 Campinas, São Paulo, Brazil

**Keywords:** *Aspergillus nidulan*, extragenic suppression, calcineurin, *CrzA*, folate biosynthesis

## Abstract

After dephosphorylation by the phosphatase calcineurin, the fungal transcription factor CrzA enters the nucleus and activates the transcription of genes responsible for calcium homeostasis and many other calcium-regulated activities. A lack of CrzA confers calcium-sensitivity to the filamentous fungus *Aspergillus nidulans*. To further understand calcium signaling in filamentous fungi and to identify genes that interact genetically with CrzA, we selected for mutations that were able to suppress *crzAΔ* calcium intolerance and identified three genes. Through genetic mapping, gene sequencing, and mutant rescue, we were able to identify these as *cnaB* (encoding the calcineurin regulatory subunit), *folA* (encoding an enzyme involved in folic acid biosynthesis, dihydroneopterin aldolase), and *scrC* (suppression of *crzA*^-^, encoding a hypothetical protein). By using a calcium indicator, Fluo-3, we were able to determine that the wild-type and the suppressor strains were either able to regulate intracellular calcium levels or were able to take up and or store calcium correctly. The increased expression of calcium transporters, *pmcA* and/or *pmcB*, in suppressor mutants possibly enabled tolerance to high levels of calcium. Our results suggest that a *cnaB* suppressor mutation confers calcium tolerance to *crzAΔ* strains through restoration of calcium homeostasis. These results stress that in *A. nidulans* there are calcineurin-dependent and CrzA-independent pathways. In addition, it is possible that CrzA is able to contribute to the modulation of folic acid biosynthesis.

To sense and respond to environmental conditions, eukaryotic cells have evolved signaling pathways to coordinate growth and metabolism. Calcium signaling is essential for several physiological responses in living organisms, such as cell cycle, differentiation, and ion homeostasis. However, unwarranted intracellular calcium concentrations can be toxic to the cell, even leading to cell death, and must be removed. Calcium binds to and activates calmodulin, a primary and highly conserved calcium-binding protein, that in turn binds to and activates effector calmodulin-dependent enzymes, such as calcineurin (Fox and Heitman 2002; Cyert 2001, 2003). The phosphatase calcineurin is a heterodimeric protein composed of the catalytic subunit A and the regulatory subunit B (Fox and Heitman 2002). The immunosuppressant cyclosporin A specifically inhibits calcineurin by mediating immunophilin binding to the phosphatase and has been used as an experimental tool to probe the function of calcineurin *in vivo* (reviewed in Liu 1993). In mammalian cells, calcineurin activates immune responses and is also involved in the cardiovascular systems (Crabtree and Olson 2002). In the *Saccharomyces cerevisiae* model system, calcineurin regulates morphogenesis, Ca^2+^ homeostasis, and stress-activated transcription (Fox and Heitman 2002; Cyert 2003). In fungi, calcineurin plays an important role in the control of cell morphology, virulence, and the action of antifungal drugs (Cruz *et al.* 2001; Fox and Heitman 2002; Cyert 2003; Steinbach *et al.* 2004, 2007; Da Silva Ferreira *et al.* 2007; Stie and Fox 2008). Inactivation of calcineurin in *Cryptococcus neoformans* affects growth at 37°, hyphal elongation during mating, and haploid fruiting (Odom *et al.* 1997; Cruz *et al.* 2000, 2002; Fox *et al.* 2001). Reduced virulence and an absence of growth in serum were observed in *Candida albicans* depleted in calcineurin activity (Cruz *et al.* 2000; Blankenship *et al.* 2003; Sanglard *et al.* 2003). In *Aspergillus fumigatus*, calcineurin inactivation decreased virulence and filamentation while also preventing growth in serum (Da Silva Ferreira *et al.* 2007; Soriani *et al.* 2008). In contrast to previous findings (Rasmussen *et al.* 1994), we found that the *Aspergillus nidulans* calcineurin catalytic subunit (calcineurin A, or CnaA) was not essential and that the *cnaA* deletion mutant shared the morphological phenotypes observed in the corresponding *A. fumigatus* mutant, *calAΔ* (Soriani *et al.* 2008).

Mammalian calcineurin dephosphorylates the nuclear factor of activated T cells (NFAT), a conserved family of transcription factors (Crabtree and Olson 2002). Calcineurin dephosphorylates NFAT by interacting with a specific domain, named the calcineurin-docking domain, which in mammalian cells has two common sequences, PxIxIT and LxVP (Aramburu *et al.* 1998, 1999; Rodriguez *et al.* 2009). Upon dephosphorylation, cytoplasmic NFAT translocates to the nucleus and activates the transcription of the genes responsible for regulating cellular calcium homeostasis (Crabtree and Olson 2002). In fungi, the best-characterized target of calcineurin is the transcription factor Crz1/CrzA that is not related to NFAT but has conserved regulatory mechanisms. An increase in cytosolic calcium results in dephosphorylation of the Crz1/CrzA transcription factor by calcineurin, thus enabling entry into the nucleus and the transcriptional activation of genes responsible for cellular calcium homeostasis (Stathopoulos-Gerontides *et al.* 1999; Karababa *et al.* 2006; Soriani *et al.* 2008; Spielvogel *et al.* 2008). The nucleocytoplasmic trafficking mechanisms of NFAT/Crz1/CrzA is the final result of the phosphorylation/dephosphorylation events and the nuclear localization/export sequences present in these transcription factors (Okamura *et al.* 2000; Polizotto and Cyert 2001; Boustany and Cyert 2002).

The *S. cerevisiae* Crz1p contains a zinc-finger DNA-binding motif that specifically binds to a 24-bp sequence in the promoter regions of genes, the calcineurin-dependent response element (Stathopoulos and Cyert 1997). *crz1Δ* mutants exhibit hypersensitivity to Mn^2+^, Li^+^, and chitosan and have defects in cellular morphology, mating, and the transcriptional response to alkaline stress (Matheos *et al.* 1997; Stathopoulos and Cyert 1997; Stathopoulos-Gerontides *et al.* 1999; Zakrzewska *et al.* 2005). Overexpression of *CRZ1* in calcineurin mutants suppressed the calcineurin deletion phenotype (Stathopoulos and Cyert 1997). *CRZ1* deletion mutants in *Schizosaccharomyces pombe* (*prz1Δ*) are hypersensitive to calcium and have reduced mRNA levels of the *PMC1* Ca^2+^ pump (Hirayama *et al.* 2003). *C. albicans* homozygotes *crz1 Δ/Δ* are moderately attenuated for virulence and sensitive to calcium, lithium, manganese, and sodium dodecyl sulfate (Onyewu *et al.* 2004; Santos and De Larrinoa 2005; Karababa *et al.* 2006). *A. fumigatus crzAΔ* strains are attenuated for virulence and have decreased conidiation, whereas their hypersensitivity to calcium and manganese has been attributed to the reduced expression of calcium transporters (Soriani *et al.* 2008). Spielvogel *et al.* (2008) have shown that the *A. nidulans crzA* deletion phenotype includes extreme sensitivity to alkaline pH, Ca^2+^ toxicity, and aberrant morphology connected with alterations of cell-wall−related phenotypes such as reduced expression of a chitin synthase gene, *chsB*.

Genes activated by Crz1/CrzA include calcium channels and transporters that import calcium into vacuoles or efflux it (Dinamarco *et al.* 2012). Besides calcium transporters, very few Crz1/CrzA targets have already been identified via microarray or chromatin immunoprecipitation sequencing strategies that have also been validated by direct demonstration. Validated genes include the *S. cerevisiae HXT3* hexose transporter (Ruiz *et al.* 2008), the *C. glabrata Yps1* glycosylphosphatidylinositol-linked aspartyl protease (Miyazaki *et al.* 2011), the *A. nidulans chsB* chitin synthase, the *Aspergillus giganteus afp* antifungal protein (Spielvogel *et al.* 2008), and the *Magnaporthe grisea CBP1* calcium binding protein (Kim *et al.* 2010). Yoshimoto *et al.* (2002) identified the *S. cerevisiae* Crz1p-binding site as 5′-GNGGC(G/T)CA-3′ by *in vitro* site selection. Recently, in *A. fumigatus*, CrzA has been shown to control directly the transcription of the *pmcA-C* calcium transporter genes by binding to their promoter regions. CrzA-binding experiments suggested that the 5′-CACAGCCAC-3′ and 5′-CCCTGCCCC-3′ sequences upstream of *pmcA* and *pmcC* genes, respectively, are possible calcineurin-dependent response element−like motifs (Dinamarco *et al.* 2012).

To further understand calcium signaling in filamentous fungi and to identify genes that interact genetically with CrzA, we selected and characterized extragenic suppressors of an *A. nidulans crzA* null mutation. We isolated mutations in three genes that, to different extents, suppressed the sensitivity of the *crzAΔ* mutant to calcium. To our knowledge, no suppressors of *crz1/crzA*-null mutations have been reported in any organism. These mutations reside within the calcineurin regulatory subunit gene *cnaB*, the *folA* gene encoding dihydroneopterin aldolase, an enzyme involved in folic acid biosynthesis (Güldener *et al.* 2004), and the *scrC* gene which encodes a protein of unknown function. Apparently these mutations suppress *crzAΔ* calcium-sensitivity by bypassing the CrzA requirement for calcium homeostasis. The identification of a mutation in *A. nidulans folA* gene suggests that CrzA is involved in the regulation of folic acid biosynthesis, adding a completely novel role for CrzA (as well as a novel role for FolA).

## Materials and Methods

### *Aspergillus* strains and growth conditions

*A. nidulans* markers and genetic methodologies were as described (Pontecorvo *et al.* 1953). The strains used in displayed experiments are listed in Table 1. Media used in this study included: complete medium YAG (2% glucose, 0.5% yeast extract, 2% agar, trace elements); and YUU, which is YAG medium supplemented with 1.2 g/L of uracil and uridine; YG medium, which has the same composition as YAG but does not contain agar and a minimal medium (MM: 1% glucose, nitrate salts, trace elements, 2% agar, pH 6.5). A modified MM with two different pHs was also used: MM pH 6.5 (1% glucose, 20 mL/L *Aspergillus* salt solution, 1% Oxoid Agar, pH 6.5 with 4 M NaOH) and MM pH 8.0 (1% glucose, 20 mL/L salt solution for pH 8.0 media, 2.7 mM NaH_2_PO_4_, 50 mM Na_2_HPO_4_, 1% Oxoid Agar, pH 8.0 with 4 M NaOH). Media were buffered with 200 mM glycolic acid, for pH 5.0, 200 mM 2-morpholinoethanesulphonic acid for pH 6.5 or 200 mM Tris-HCl for MM for pH 8.0. The composition of the trace elements, vitamins, and nitrate salts are described in the appendix to Käfer (1977). Standard genetic techniques for *A. nidulans* (Käfer 1977) were used for all constructions. For the heat-sensitive (hs^−^) suppressor mutants, the permissive and restrictive temperatures for growth were 30° and 44°, respectively.

**Table 1:** *Aspergillus nidulans s*trains used in display items

Strains	Genotypes	References
TNO2A3	*pyroA4*; *pyrG89*; *argB2*; *nkuAΔ*::*argB*	Nayak *et al.* 2006
R21	*pabaA1*; *yA2*	FGSC A234
GR5	*pyrG89*; *wA3*; *pyroA4*	FGSC A773
UI224	*argB*; *pyrG89*; *yA2*	FGSC
BER02	*pyroA4*; *pyrG89*; *argB2*; *nkuAΔ*::*argB*; *crzAΔ*::*pyr-4*	Spielvogel *et al.* 2008
*cnaAΔ*	*pyroA4*; *pyrG89*; *wA3*; *cnaAΔ*::*pyroA*	Soriani *et al.* 2008
*crzAΔ*	*pyroA4*; *pyrG89*; *argB2*; *nkuAΔ*::*argB*; *crzAΔ*::*pyroA*	This work
*alcA*::*crzA cnaAΔ*	*pyroA4*; *pyrG89*; *argB2*; *nkuAΔ*::*argB*; *cnaAΔ*::*pyroA*; *alcA*::*crzA*::*pyr-4*	This work
*rcnAΔ*	*pyroA4*; *pyrG89*; *argB2*; *nkuAΔ*::*argB*; *rcnAΔ*::*pyrG*	Soriani *et al.* 2010
*cnaB1 crzAΔ*	*pyroA4*; *pyrG89*; *argB2*; *nkuAΔ*::*argB*; *crzAΔ*::*pyr-4*; *cnaB1*	This work
*folA1^-^ crzAΔ*	*pyroA4*; *pyrG89*; *argB2*; *nkuAΔ*::*argB*; *crzAΔ*::*pyr-4*; *folA1*	This work
*cnaB1*	*pyroA4*; *pyrG89*; *argB2*; *wA3*; *nkuAΔ*::*argB*; *cnaB1*	This work
*folA1*	*pyroA4*; *pyrG89*; *argB2*; *wA3*; *nkuAΔ*::*argB*; *folA1*	This work
rev-1	*crzAΔ*::*pyr4*; *scrC3*; *paba*-; *pantoB100*; *wA3*	This work
rev-2	*crzAΔ*::*pyr4*; *cnaB2*; *paba*-; *wA3*	This work
rev-3	*crzAΔ*::*pyr4*; *scrC4*; *paba*-; *pantoB100*; *wA3*	This work
*scrCΔ*	*pyroA4*; *pyrG89*; *argB2*; *nkuAΔ*::*argB*; *scrCΔ*::*pyroA*	This work
*cnaB1 crzAΔ*	*pyroA4*; *pyrG89*; *argB2*; *cnaB1 crzAΔ*	This work
*cnaBΔ*	*cnABΔ*::*pyroA*	This work
*cnaBΔ crzAΔ*	*cnaBΔ*::*pyroA crzAΔ*::*pyrG*	This work

### Mutagenesis and the selection of suppressors

For mutations now designated *cnaB2*, *scrC3*, and *scrC4*, *A. nidulans* conidiospores, from cultures grown on *Aspergillus* complete solid medium were exposed to 254 nm ultraviolet light with a UV Stratalinker 1800 (Stratagene, UK) for approximately 10 min. The subsequent steps were performed in the dark. Conidial suspensions were made in sterile water plus 0.1% (w/v) Tween 80 and spread directly onto supplemented MM containing 200 mM CaCl_2_. Plates were then wrapped in foil and incubated at 30° for 3 d, after which the foil was removed and the plates were incubated for an additional 7 d at 30°. For mutations now designated *cnaB1* and *folA1*, conidiospores were treated with 4-nitroquinoline-1-oxide: 1.0 mL of phosphate-buffered saline (PBS: NaCl, 8.01 g/L; KCl, 0.20 g/L; Na_2_HPO_4_•2 H_2_O, 1.78 g/L; KH_2_PO_4_, 0.27 g/L, pH 7.4) containing 1.5 µg/mL 4-nitroquinoline-1-oxide and 2 × 10^7^ conidiospores/mL was incubated for 30 min at 30° with agitation (250 rpm, 1000 g). After this period, 1 mL of sodium thiosulfate (5% w/v) was added to stop the mutagenesis. This solution was centrifuged (4000 rpm for 5 min), the pellet was appropriately diluted and plated on YUU medium. Approximately 90% of the conidia were killed by this treatment. Aliquots of the conidial suspension (0.1 mL) were spread on YUU + 300 mM CaCl_2_ plates that were incubated at 30° for 3 d. Suppressors were isolated and subsequently retested for growth at 30 and 44°.

### Staining and microscopy

For light microscopy experiments, strains were grown on sterile glass coverslips within a Petri dish overlaid with the appropriately supplemented liquid YG medium containing ~10^6^ conidiospores/mL of the relevant strain. Conidia settled to the bottom of the dish and adhered tightly to the coverslips during germination.

To stain the nuclei of adherent germlings, after 12-hr incubation at 28 or 44°, coverslips were immersed in fixative (3.7% formaldehyde, 50 mM sodium phosphate buffer pH 7.0, 0.2% Triton X-100) for 30 min at room temperature. Then, briefly rinsed with PBS buffer (140 mM NaCl, 2 mM KCl, 10 mM NaHPO_4_, 1.8 mM KH_2_PO_4_, pH 7.4) and incubated for 5 min with 100 ng/mL DAPI (4′,6-diamino-2-phenyylindole; Sigma Chemical Co.) and 100 ng/mL calcofluor white (Fluorescent brightener; Sigma Chemical Co.). After staining, the germlings were washed with PBS buffer for 5 min at room temperature, rinsed with distilled water, and mounted in Citifluor. The samples were finally examined using a Zeiss epifluorescence microscope.

The reciprocal shift experiments, used to determine the stage of interphase at which the *folA1* strain arrested, were performed as described (Bergen *et al.* 1984). Conidia from the *folA1* strain were inoculated on coverslips as described previously and incubated at 44° for 7 hr. Untreated germlings were either fixed (as a control), transferred to 44° for 3 hr or to YG plus 25 mM hydroxyurea (HU) for 10 min (at 44°), and then shifted to 28° with HU for 3 hr. For the reciprocal experiments, conidia were arrested in S-phase by inoculating onto coverslips in rich media containing 25 mM HU and incubated for 7 hr at 28°. The coverslips were either fixed (as a control) or transferred to 28° YG medium in the absence of HU or shifted to 44° without HU. Coverslips were stained as described above and the number of nuclei per germling determined.

### Determination of intracellular calcium levels

A fluorescent calcium indicator (Fluo-3, AM; Molecular Probes, F14218) was used to investigate the maintenance of intracellular calcium homeostasis. Strains were grown for 16 hr at 30° in 1 mL of YUU broth in a 24-well plate containing a circular coverslip (12 mm diameter) per well. After incubation, the samples were washed twice with 1 mL of PBS after which 1 mL of 200 mM CaCl_2_ containing 1 µM Fluo-3 was added and incubated for 10 min at 30°. The coverslips were then washed three times with 1 mL of water and mounted on a slide with 5 µL of 50% glycerol. Control samples were incubated for 10 min with Fluo-3 without calcium treatment. Germlings were observed using a fluorescence microscope (Observer.Z1, Zeiss). To allow comparisons between strains, the same exposure time and light intensity were applied to all samples.

### Molecular techniques

Standard genetic techniques for *A. nidulans* were used for all strain constructions (Kafer 1977). DNA manipulations were performed according to Sambrook and Russell (2001). All polymerase chain reactions (PCRs) were performed using High Fidelity Platinum Taq DNA Polymerase (Invitrogen). The *scrC* (AN8823) deletion cassette used for DNA-mediated transformation was constructed by *in vivo* recombination in *S. cerevisiae* as described by Colot *et al.* (2006). In summary, a 1.5-kb region either side of the open reading frame (ORF) was selected for primer design. The primer pairs were named 5F and 5R for the 5′-untranslated region (UTR) and 3F and 3R for the 3′-UTR. The 5F and 3R primers contained a short sequence homologous to the MCS of the pRS426 plasmid (Supporting Information, Table S1). Both the 5′- and 3′-UTR fragments were PCR-amplified from genomic DNA extracted from *A. nidulans* strain A4. The *pyrG* gene, used as selection marker for transformation, was amplified from the pCDA21 plasmid (Chaveroche *et al.* 2000). Deletion cassette generation was achieved by transforming the three fragments for each construction, along with the *Bam*HI/*Eco*RI-cut pRS426 plasmid, into the *S. cerevisiae* strain SC94721 using the lithium acetate method (Schiestl and Gietz 1989). Yeast transformant DNA was extracted (Goldman *et al.* 2003), dialyzed, and transformed by electroporation into *Escherichia coli* strain DH10B, to rescue the pRS426 plasmid. The deletion cassette was subsequently PCR-amplified from the plasmid and used for *A. nidulans* transformation (Osmani *et al.* 1987). Transformants were selected for the ability to grow on MM in the absence of uridine and uracil. For the *cnaB*^G120D^ gene replacement experiment, the *cnaB*^G120D^ allele was PCR-amplified from a *cnaB1* strain and DNA fragments transformed into a wild type strain. Transformants were selected at 44°. Southern blot analyses were used throughout to demonstrate that transformation cassette had integrated homologously at the targeted *A. nidulans* locus.

### RNA isolation and RT-PCRs

Liquid YG cultures inoculated with 1.0 × 10^8^ mL^−1^ conidia were incubated in a reciprocal shaker at 30° for either 8 or 12 hr. Mycelia were aseptically transferred to fresh YG medium in the absence or presence of 200 mM CaCl_2_ for 10 and 30 min. The mycelia were harvested by filtration, washed with sterile distilled water, immediately frozen in liquid nitrogen, and ground into a powder while frozen. Total RNA was extracted with Trizol (Life Technologies) and RNAse-free DNAse treated as previously described by Semighini *et al.* (2002).

All PCRs were performed using an ABI 7500 Fast Real-Time PCR System (Applied Biosystems) and Taq-Man Universal PCR Master Mix kit (Applied Biosystems). The reactions and calculations were performed according to Semighini *et al.* (2002). The primers and Lux fluorescent probes (Invitrogen) used in this work are described in Table S1.

### Modeling of calcineurin

Protein homologs were identified by a BLAST search on the Uniprot database (www.uniprot.org). Comparative modeling of the regulatory subunit of calcineurin was performed using the X-ray structure deposited with the Protein Data Bank (PDB) code 2P6B as template. This corresponds to the human calcineurin (Li *et al.* 2007). The unitary crystal cell of this structure contains two copies of the catalytic subunit of calcineurin bound to their corresponding regulatory proteins plus a peptide carrying the sequence PVIVIT. Only the regulatory subunit bound to calcium ions chain labeled as chain D in this structure was used in the modeling. The model comprises residues 26 to 135, which includes all the four EF hand motifs. The template and target sequences aligned without gaps with 65% identity. A comparative model was constructed using MODELER (Sali *et al.* 1995). The resulting model contained more than 90% of residues in the most favored regions of the Ramachandran plot and no residues in the disallowed regions. Molecular graphics were rendered with VMD (Humphrey *et al.* 1996).

## Results

### Selection and characterization of crzAΔ suppressors

**Figure 1:**
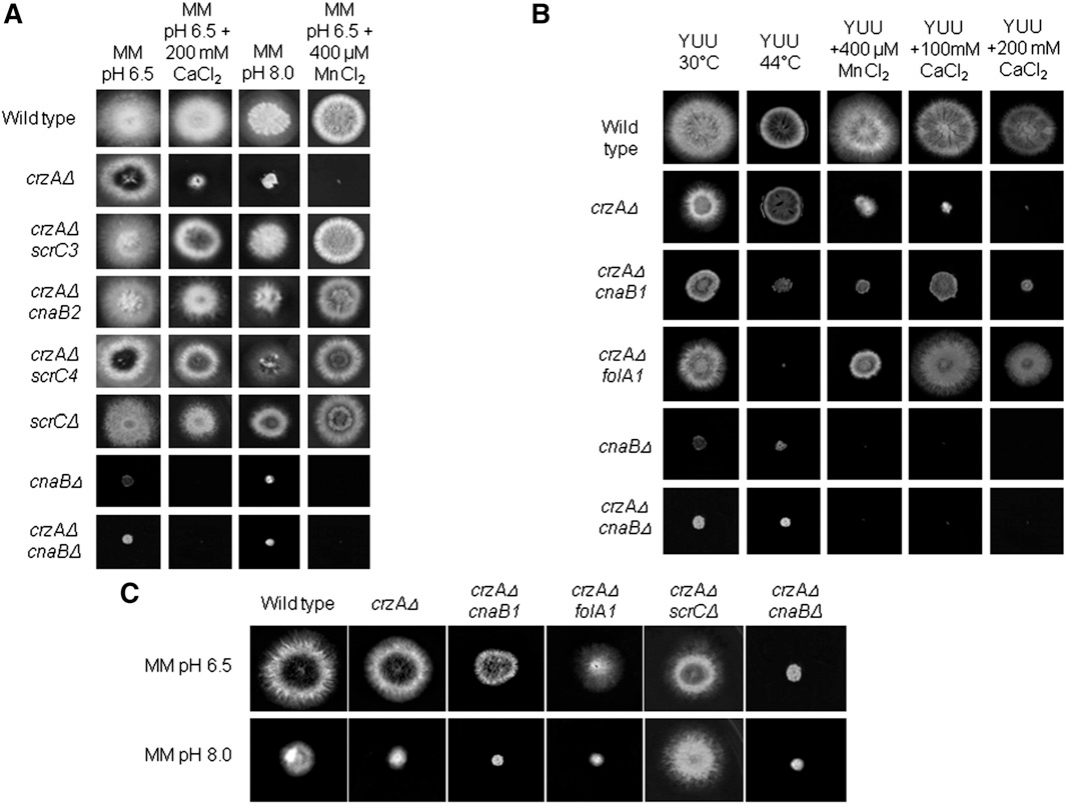
Growth phenotypes of the *ΔcrzA* extragenic suppressors. (A) Wild-type, *crzAΔ*, *crzAΔ scrC3*, *crzAΔ cnaB2*, *crzAΔ scrC4*, *scrCΔ*, *cnaBΔ*, and *crzAΔ cnaBΔ* mutant strains were grown on MM (pH 6.5), MM+200 mM CaCl_2_ (pH 6.5), or MM (pH 8.0) for 72 hr at 30° or MM+400 uM MnCl2 (pH 6.5) for 72 hr at 44°. (B) Wild-type, *crzAΔ*, *crzAΔ cnaB1*, *crzAΔ folA1*, *cnaBΔ*, *crzAΔ cnaBΔ* mutant strains were grown either on YUU 30°, YUU 44°, YUU+400 μM MnCl_2_, YUU+100 mM CaCl_2_, or YUU+200 mM CaCl_2_ for 72 hr. (C) Wild-type, *crzAΔ*, *crzAΔ cnaB1*, and *crzAΔ folA1* mutant strains were grown either on MM (pH 6.5) or MM (pH 8.0) for 72 hr at 30°. Except where otherwise indicated, the growth temperature was 30°.

To gain insight into possible targets of the CnaA-CrzA regulatory system, we performed a suppression analysis of a *crzAΔ* mutant. Two strategies were used to identify suppressors of the *crzAΔ* mutation: (1) the mutations now designated *cnaB2*, *scrC3*, and *scrC4* were selected as suppressors of calcium toxicity (up to 200 mM CaCl_2_) at 37° and the recovery of the poor growth phenotype observed for the *crzAΔ* strain grown on pH 8 medium (growth at 30° is shown in Figure 1A); and (2) the mutations now designated *cnaB1* and *folA1* were selected for as suppressors of calcium toxicity (up to 100 or 200 mM CaCl_2_) at 30° and screened for an inability to grow at 44° (hs^−^ phenotype). The MM was used in these experiments allowed the precise adjustment of pH. However, the observed phenotypes for these mutants were identical in either MM or YUU (Figure 1B and data not shown). The segregants from the crosses between *crzAΔ cnaB1* x wild type strain GR5 and *crzAΔ folA1* x GR5 were analyzed to determine whether the conditional hs^-^ mutation in the suppressors cosegregated with the *scr* mutations. The presence of calcium resistant (Ca^R^), hs^+^ progeny would have signified a lack of cosegregation between the *scr* mutation and the conditional mutation (Table S2). However, thermo-sensitivity and calcium resistance in these two mutants cosegregated (see Figure 1B). Neither of these two mutations improves growth of *crzAΔ* strains at pH 8 (Figure 1C). Deletion of *cnaB* leads to poor growth on all media but does not lead to appreciable thermo-sensitivity and/or improve growth of *crzAΔ* strains on calcium-containing or pH 8 media (Figure 1). In addition to calcium sensitivity, *crzAΔ* strains are hypersensitive to manganese toxicity (Figure 1B). The *cnaB2*, *folA1*, *scrC3*, and *scrC4* mutations but neither *cnaB1* nor *cnaBΔ* confer manganese resistance (Figure 1B).

**Figure 2:**
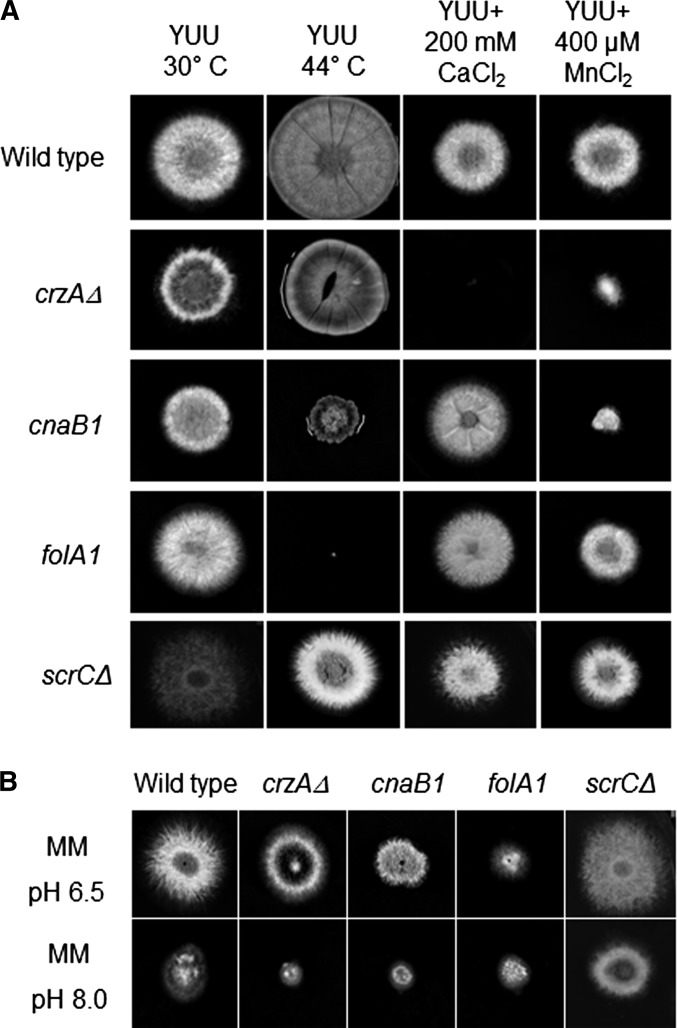
Growth phenotypes of the *cnaB1* and *folA1* mutations. (A) Wild-type, *crzAΔ*, *cnaB1*, *folA1*, and *scrCΔ* mutant strains were grown either on YUU 30°, YUU 44°, YUU+200 mM CaCl_2_, or YUU+400 μM MnCl_2_ for 72 hr. (B) Wild-type, *crzAΔ*, *cnaB1*, *folA1*, and *scrCΔ* mutant strains were grown either on MM (pH 6.5) or MM (pH 8.0) for 72 hr at 30°.

**Figure 3:**
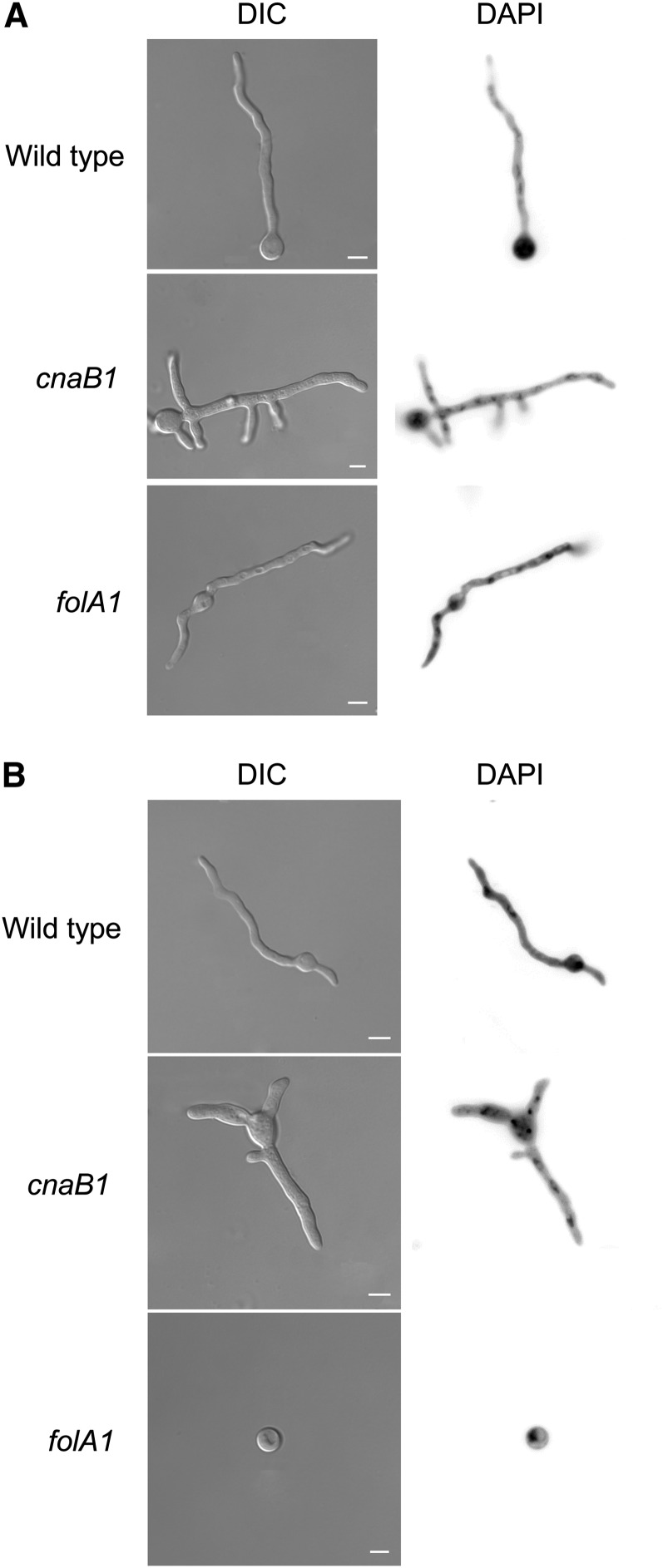
Morphology and numbers of nuclei in the wild-type, *cnaB1*, and *folA1* mutant strains. (A) The wild-type, *cnaB1*, and *folA1* mutant strains were grown at 30° for 16 hr and stained with DAPI. (B) The wild-type, *cnaB1*, and *folA1* mutant strains were grown at 44 °C for 16 hr and stained with DAPI. Bars, 10 µm.

In outcrossed *crzA^+^* backgrounds, *cnaB2* and the *scrC* mutations do not have discernible phenotypes. *cnaB1* and *folA1*; however, continue to show thermosensitivity and *cnaB1* confers clear manganese and pH 8 hypersensitivity (Figure 2, A and B). *cnaB1* colonies at both permissive and restrictive temperatures are wrinkled and produce a dark pigment at their peripheries (Figures 1B and 2A and data not shown). When *cnaB1* germlings were incubated at 30° or 44° for 16 hr and stained with DAPI, the numbers of nuclei at the two temperatures were about the same (8.84 ± 0.08 at 30° *vs.* 7.77 ± 0.22 at 44° for the wild type and 9.21 ± 0.06 at 30° *vs.* 8.09 ± 0.22 at 44° for the *cnaB1*); however, virtually all the germlings showed increased branching (Figure 3). When *folA1* germlings were incubated at 30° or 44° for 16 hr and stained with DAPI, the number of nuclei at 30° was comparable with that of wild type germlings (8.84 ± 0.07 at 30°; Figure 3A). At the restrictive temperature, however, 60% of the conidiospores had a single nucleus (Figure 3B), in contrast to the wild-type germlings that normally undergo two or three nuclear divisions under these conditions (Figure 3B). These results suggest that the *cnaB1* mutation affects branching frequency, whereas the *folA1* mutation affects the nuclear division cycle. Up to approximately 12 hr of incubation at 44°, the *folA1* block is reversed by shifting to 28°.

Reciprocal shift assays were performed to determine the stage of interphase at which arrest occurs in the *folA1* mutant (Table 2). When conidia were inoculated into YG medium and incubated for 7 hr at 44°, we again observed that only 60% of nuclei underwent mitotic division (Table 2). When conidia were inoculated into YG medium and incubated for 7 hr at 44° then shifted to YG medium at 28° in either the presence or absence of 25 mM HU for 3 hr, approximately 60% of nuclei underwent mitotic division, regardless of the presence of HU (Table 2). This finding suggests that the cells exit from M phase, arrest at 44°, traverse mitosis and G_1_ phase, and arrest in S phase in the presence of HU. As a control, the reciprocal experiment was performed, in which conidia were inoculated in YG medium at 28° either in the presence or absence of 25 mM HU for 7 hr and then shifted to YG medium at 28° or 44° for 3 hr. After shifting to 28°, approximately 30% of the nuclei without HU underwent mitotic division (Table 2). However, approximately 20% of the germlings completed one nuclear division after shifting to 28° plus HU (Table 2). These results indicate that the *folA1* mutant is blocked in M phase at the restrictive temperature.

**Table 2:** Double reciprocal shift assay of a *folA1* strain

Treatment*^a^*	Initial Conditions		Shift		Percent with Two Nuclei*^b^*
	Temperature	Time, hr	Temperature	Time, hr	
Downshifts					
A	44°	7	No shift	0	54 ± 1.7
B	44°	7	28°	3	55 ± 4.6
C	44°	7	28° + 25 mM HU*^c^*	3	46 ± 2.9
Upshifts					
D	28° + 25 mM HU	7	No shift	0	0
E	28° + 25 mM HU	7	28°	3	27 ± 3.1
F	28° + 25 mM HU	7	44°	3	21 ± 2.4

HU, hydroxyurea.

a One hundred germlings were counted for each experiment and the percentage having one nuclear division was determined.

b Percentage of cells completing at least one nuclear division.

c Before transferring the germlings to the treatment C (28° + HU), the germlings were incubated in the presence of 25 mM HU for 10 min at 44°.

**Figure 4:**
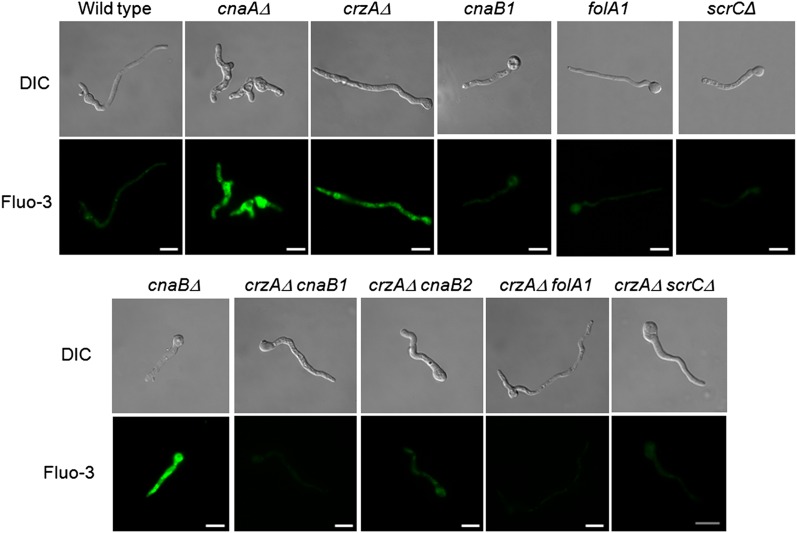
The *cnaB1*, *cnaB2*, *folA1*, and *scrCΔ* mutations reduce cytosolic calcium levels in a *crzAΔ* background. Strains having the indicated relevant, partial genotypes shown were grown in liquid YUU for 16 hr at 30° and then exposed to 200 mM CaCl_2_ for 30 min followed by 10 min exposure to Fluo-3. Bars, 10 µm.

Calcium accumulation was determined in the wild-type, *cnaAΔ*, *crzAΔ*, *cnaB1*, *folA1*, *scrCΔ*, and several suppressed *crzAΔ* double-mutant strains using the Fluo-3 calcium indicator, a highly sensitive dye for rapid measurement of calcium levels in cells. Germlings of the *cnaAΔ*, *cnaBΔ*, and *crzAΔ* mutant strains exposed to CaCl_2_ 200 mM for 10 min, fluoresced strongly (Figure 4). In contrast, the wild-type and suppressor mutant strains had much reduced fluorescence as did the suppressed *crzAΔ* double mutant strains (Figure 4). These results suggest that the wild-type and the suppressor strains are either able to regulate intracellular calcium levels or that calcium is not taken up and/or stored correctly.

To address the possibility that the suppression of the *crzAΔ* calcium sensitivity might be related to the altered expression of calcium transporters, we investigated the mRNA accumulation of two *A. nidulans PMC1* homologs, *pmcA* (AN1189.3) and *pmcB* (AN4920.3). The putative calcium-transporting vacuolar ATPases, PmcA-B, are involved in calcium homeostasis and are transcriptionally induced in response to CaCl_2_ in a CrzA-dependent manner (Hagiwara *et al.* 2008). Using reverse transcription quantitative PCR analysis, we quantified the mRNA accumulation of these genes in the wild type, *crzAΔ*, *crzAΔ cnaB1*, *crzAΔ folA1*, and *crzAΔ scrC3* strains in response to short pulses of 200 mM CaCl_2_ (Figure 4). In the wild-type strain, *pmcA* and *pmcB* mRNA accumulation increased after exposure to CaCl_2_ for 10 and 30 min, with the latter showing a greater increase, whereas this accumulation was completely abolished for all transporters in the *crzAΔ* mutant strain (Table 3). The reduced expression of these ion pumps in the *crzAΔ* background may explain the observed calcium-sensitivity of this strain. Similar to the wild-type strain, all the suppressor mutant strains demonstrated increased *pmcA* and/or *pmcB* mRNA accumulation post exposure to calcium, unlike the *crzAΔ* mutant (Table 3). Interestingly, in the *crzAΔ cnaB1* mutant strain *pmcA* demonstrated a faster increase in mRNA accumulation than the wild-type strain (Table 3). In addition, the *crzAΔ scrC3* mutant demonstrated a far greater level of *pmcA* mRNA accumulation than the wild type strain. Collectively these results show that the suppressor mutants were able to induce calcium transporters in response to calcium exposure, unlike the *crzAΔ* strain, thus possibly contributing to the ability to cope with high calcium levels.

**Table 3:** Quantitative measurements by RT-qPCR of the *pmcA*, *-B* mRNA accumulation when the wild-type and mutant strains were exposed to a short pulse of CaCl_2_ (10 and 30 min)

	*pmcA* Control	*pmcA*, 10 min	*pmcA*, 30 min	*pmcB* Control	*pmcB*, 10 min	*pmcB*, 30 min
Wild type	0.24 ± 0.01	1.62 ± 0.25 (6.8 X)	2.85 ± 0.41 (12.0 X)	0.01 ± 0.001	0.16 ± 0.02 (15.5 X)	0.36 ± 0.02 (35.3 X)
*crzAΔ*	0.91 ± 0.02	0.87 ± 0.21 (0.9 X)	0.88 ± 0.24 (0.9 X)	0.03 ± 0.001	0.05 ± 0.001 (0.6 X)	0.05 ± 0.001 (1.6 X)
*crzAΔ cnaB1*	0.60 ± 0.03	8.37 ± 0.97 (13.9 X)	3.40 ± 0.01 (5.7 X)	0.01 ± 0.00	0.03 ± 0.005 (2.0 X)	0.04 ± 0.005 (4.0 X)
*crzAΔ folA1*	0.17 ± 0.01	0.70 ± 0.06 (4.2 X)	0.81 ± 0.01 (4.8 X)	0.009 ± 0.00	0.04 ± 0.004 (4.1 X)	0.16 ± 0.002 (18.4 X)
*crzAΔ scrC3*	0.22 ± 0.002	3.00 ± 0.30 (13.9 X)	13.1 ± 1.50 (60.7 X)	0.12 ± 0.001	0.47 ± 0.01 (3.8 X)	0.16 ± 0.01 (1.3 X)

Observations: (1) Control: strains were grown for 16 hr in YG medium at 37°; (2) the values represent the [*pmcA* cDNA]/[*tubC* cDNA] and [*pmcB* cDNA]/[*tubC* cDNA]. Values between parentheses correspond to the number of times the *pmcA* and *pmcB* mRNA accumulated comparatively to the control.

### Two crzAΔ suppressor mutations lie in cnaB, encoding the regulatory subunit of calcineurin

**Figure 5:**
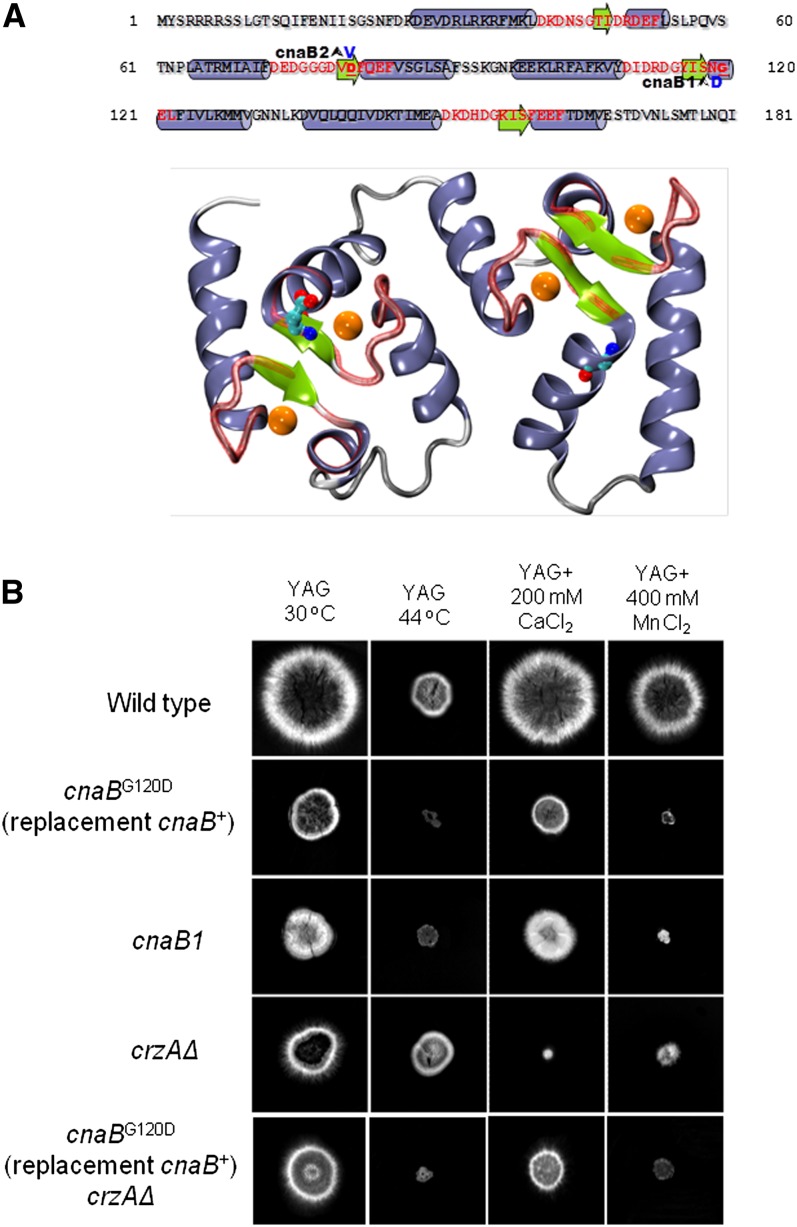
Structural modeling of CnaB and verification that cnaB1 is responsible for the observed phenotype. (A) Top: Primary sequence of CnaB. Mutationally altered residues (Asp81Val and Gly120Asp of CnaB2 and CnaB1, respectively) are in bold blue letters. Red letters indicate residues in EF hand motifs. The predicted secondary structure features are indicated by background blue cylinders and green arrows for alpha helices and beta strands, respectively. Bottom: Illustration of the homology model of CnaB obtained from the crystal structure of human calcineurin (Li *et al.* 2007; PDB id 2P6B). The color scheme is identical to that described previously. The putative positions of calcium ions within the EF hands are indicated by orange spheres. The positions of Asp81 and Gly120 are indicated by red balls on green sticks. (B) Wild-type, *cnaB*^G120D^ (*cnaB*^+^ was replaced with *cnaB*^G120D^), *cnaB1*, *crzAΔ*, and a double *cnaB*^G120D^ (*cnaB*^+^ was replaced with *cnaB*^G120D^) *crzAΔ* were grown either on YAG at 30°, YAG at 44°, YAG+200 mM CaCl_2_ at 30°, or YAG+400 μM MnCl_2_ for 72 hr.

The mutations now designated *cnaB1* and *cnaB2* were mapped to the vicinity of *cnaB* in linkage group I (File S1). Intriguingly, *cnaB1* germlings are wider than wild-type germlings (74.4 ± 6.2 μm for *cnaB1* mutant and 49.1 ± 5.6 μm for the wild-type strain; Figure 3), similar to *cnaAΔ* germlings (Soriani *et al.* 2008). We therefore sequenced the *cnaB* gene from the *cnaB1* and *cnaB2* strains. *cnaB1* resulted in a G120D mutation (mutant codon GAC) and *cnaB2* resulted in a D81V mutation (mutant codon GTT) (Figure 5A).

To confirm that the *cnaB1* and *-2* mutations were responsible for the observed phenotype, the *cnaB*^G120D^ mutation (the same as *cnaB1*) was independently constructed by gene replacement. Figure 5B shows that the *cnaB*^G120D^ gene-replacement allele leads to very similar phenotypes to the *cnaB1* mutation. Construction of (gene replacement) *cnaB*^G120D^ *crzAΔ* double mutants showed that calcium toxicity is suppressed (Figure 5B).

To gain structural insight into the possible effects of the *cnaB* mutational changes, we constructed a homology model of the regulatory subunit of calcineurin. The mutated residues are in the second and third EF hands of the protein, respectively (Figure 5A). Inspection of the structural models indicates that both mutant residues are in close proximity to the calcium binding sites and fully exposed to the solvent. These mutations are therefore unlikely to result in steric clashes. Conservation analysis (not shown) shows that although both positions are well-conserved, hydrophobic amino acids are present at position 81 in homologous proteins (*e.g.*, tyrosine, alanine and isoleucine, in the proteins Q9D869, Q5DFV5 and E6ZKS8 at the UniProt database, respectively). This suggests that both substitutions can be tolerated without significant structural distortions.

Examination of the structural model (Figure 5A) suggests that the D81V substitution reduces the negative charge of the second EF hand near to the calcium binding site. Although there is no shortage of acidic residues for calcium coordination in the second EF hand (consider the neighboring Asp73, Glu74, Asp75, Asp79, and Glu84), the reduction of the negative charge might reduce affinity of this site for calcium.

The interpretation of the possible effect of the G120D substitution is less straightforward. One possibility is that the presence of an aspartic acid at position 120 might distort the third EF hand through electrostatic repulsion or altered hydrogen bonding positions (Figure 5A). Of note, however, is a common feature in the structures of the homologous calcineurin heterodimeric structures reported in the PDB (www.ncbi.nlm.nih.gov): the backbone nitrogen of G120 forms a hydrogen bond with a conserved acidic residue in the catalytic subunit of calcineurin (Figure S1). This acidic residue corresponds to Glu35 in *A. nidulans* CnaA. Introduction of a negatively charged side chain at position 120 might disrupt the normal hydrogen bonding and replace it with strong electrostatic repulsion, putatively reducing the binding energy between the catalytic and regulatory subunits. Thus, molecular modeling suggests that both mutations would result in a higher calcium concentration threshold for activation of the catalytic subunit of calmodulin. The effects of these mutations on the cooperative conformational changes of the calcineurin regulatory subunit are currently unclear. Taken together, these results suggest that these two mutational changes in EF-hand domains are able to suppress the *crzAΔ* calcium toxicity by increasing calcineurin activity.

### A mutation in folA of the folic acid biosynthetic pathway can suppress crzAΔ calcium toxicity

The mutation now designated *folA1* was located to the linkage group III region between AN4957 (*galE*) and AN4998 (*gapA*; see File S1). Twenty-seven of the 44 ORFs in this region were sequenced, enabling the identification of a nucleotide change in AN4979. The translation product of AN4979 shows high similarity to the dihydroneopterin aldolase [EC:4.1.2.25 catalyzing the conversion of 7,8-dihydropterin to 6-hydroxy-methyl-7,8-dihydropterin for the synthesis of tetrahydrofolate (Guldener *et al.* 2004)] components of *S. cerevisiae* Fol1p. An alignment showing sequence conservation between FolA and some of its homologs, demonstrating a possible structural basis for the *folA1* mutant phenotype, is shown in Figure S2. In the *folA1* allele a G to A nucleotide change at position 52 results in D18N. This mutational change occurs in the first FolA Tunnelling fold (T-fold; pfam 02152).

**Figure 6:**
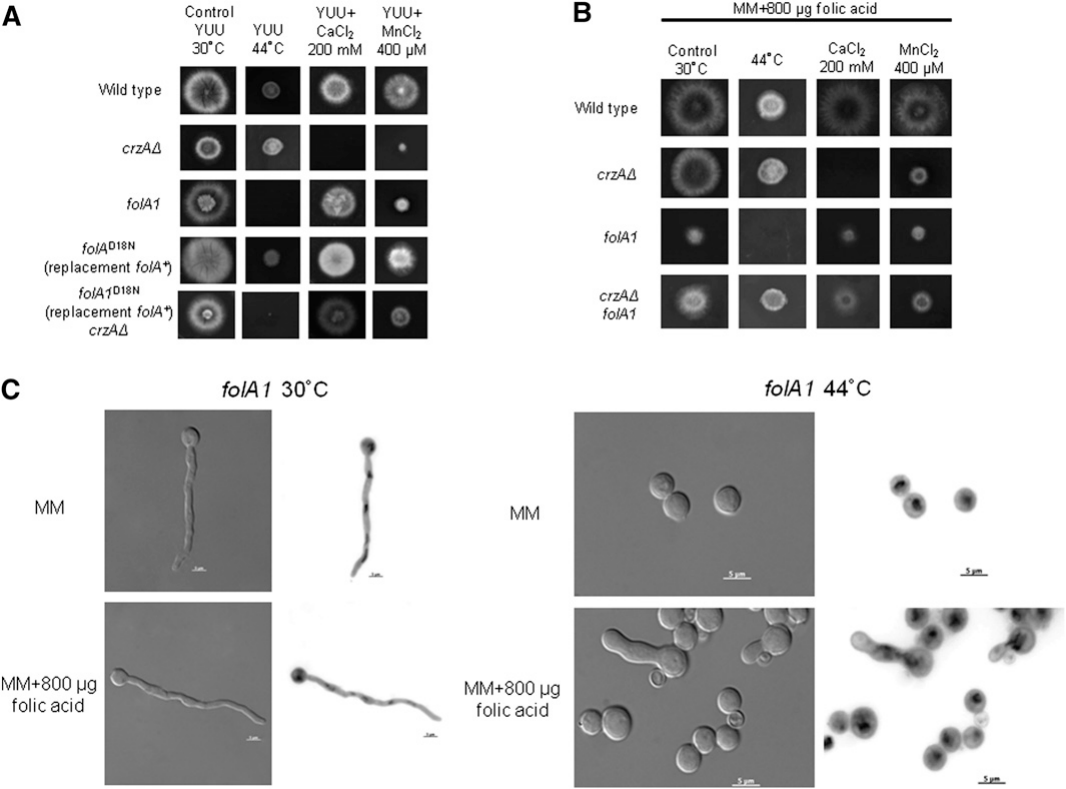
The *folA1* mutation cannot be adequately supplemented by folic acid. (A) Wild-type, *crzAΔ*, *folA1*, *folA*^D18N^ (*folA*^+^ was replaced with *folA*^D18N^), and a double *folA*^D18N^ (*folA*^+^ was replaced with *folA*^D18N^) *crzAΔ* mutant strains were grown either on YUU at 30°, YUU at 44°, YUU+200 mM CaCl_2_ at 30°, or YUU+400 μM MnCl_2_ at 30° for 72 hr; (B) Wild-type, *crzAΔ*, *crzAΔ folA1*, and *crzAΔ folA1* were grown on MM+800 μg/ml folic acid at 30°, MM+800 μg/mL folic acid at 44°, MM+800 μg/mL +200 mM CaCl_2_ at 30°, or MM+800 μg/mL folic acid+400 μM MnCl_2_ at 30 °C for 72 hr; and (C) A *folA1* strain was grown at either 30° or 44° for 24 hr in MM with or without supplementation with 800 µg of folic acid and stained with DAPI. Panels at left: DIC; panels at right: DAPI-stained in inverted contrast. Bars, 5 µm.

The identity of the *folA1* mutation as that responsible for suppression of *crzAΔ* calcium toxicity and thermo-sensitive growth was established by the ability of the *folA^+^* allele, introduced by transformation, to rescue the *folA1* phenotype (Figure 6A) and the ability of the *folA*^D18N^ allele, introduced by gene replacement of the *folA^+^* allele, to result in thermosensitivity and, when crossed into a *crzAΔ* background, to alleviate calcium toxicity (Figure 6A). The addition of 1.8 mM folic acid does not improve growth of *folA1* strains at 44°, nor does it improve their growth at 30° (Figure 6, B and C). Nevertheless, when a *folA1* strain was grown at 44° in the presence of 1.8 mM folic acid, approximately 15% of the germlings released germ tubes and the nuclei behaved as if in anaphase (Figure 6D). As the degree to which *A. nidulans* takes up folic acid is uncertain, the interpretation of these attempts at folic acid supplementation is unclear.

### Mutations in scrC can suppress crzAΔ calcium toxicity

**Figure 7:**
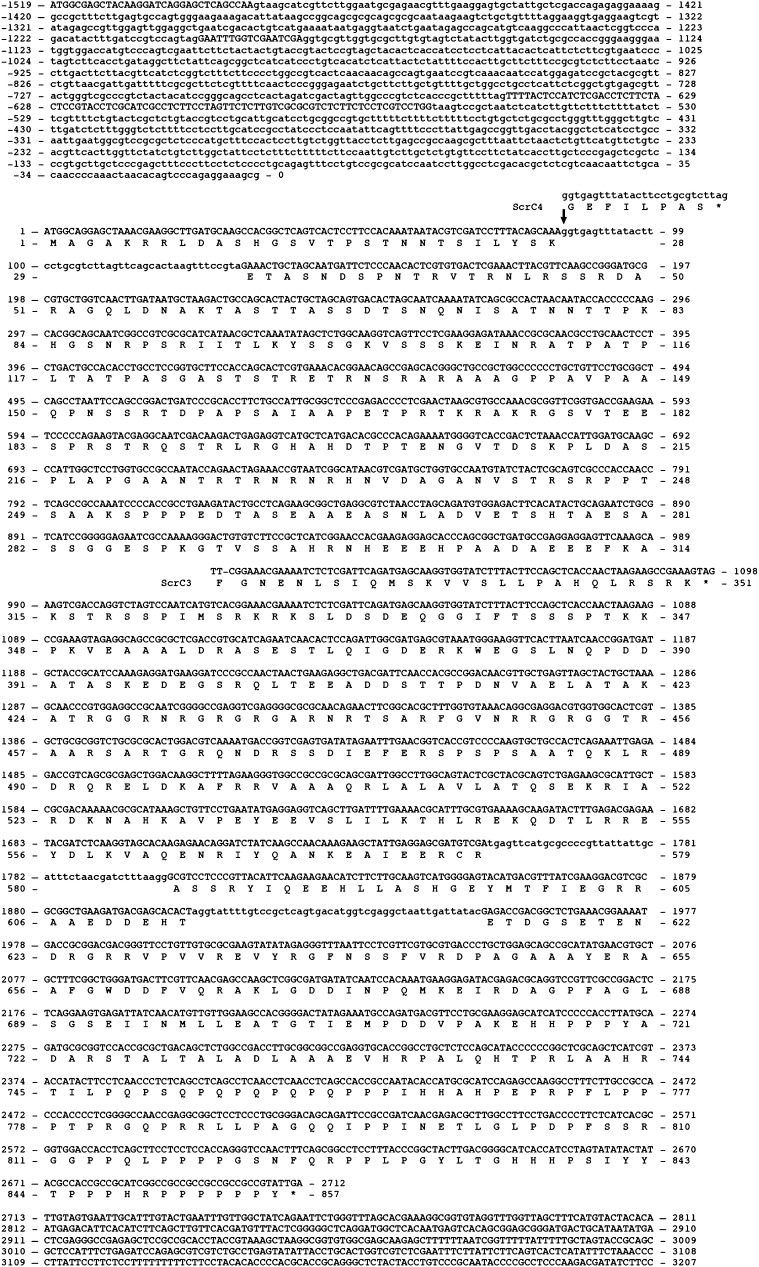
Nucleotide and derived amino acid sequences of *scrC* (AN8823). Intron sequences are shown in lower case letters. Nucleotide and translation product changes associated with the *scrC3* and -*4* mutations are shown.

**Figure 8:**
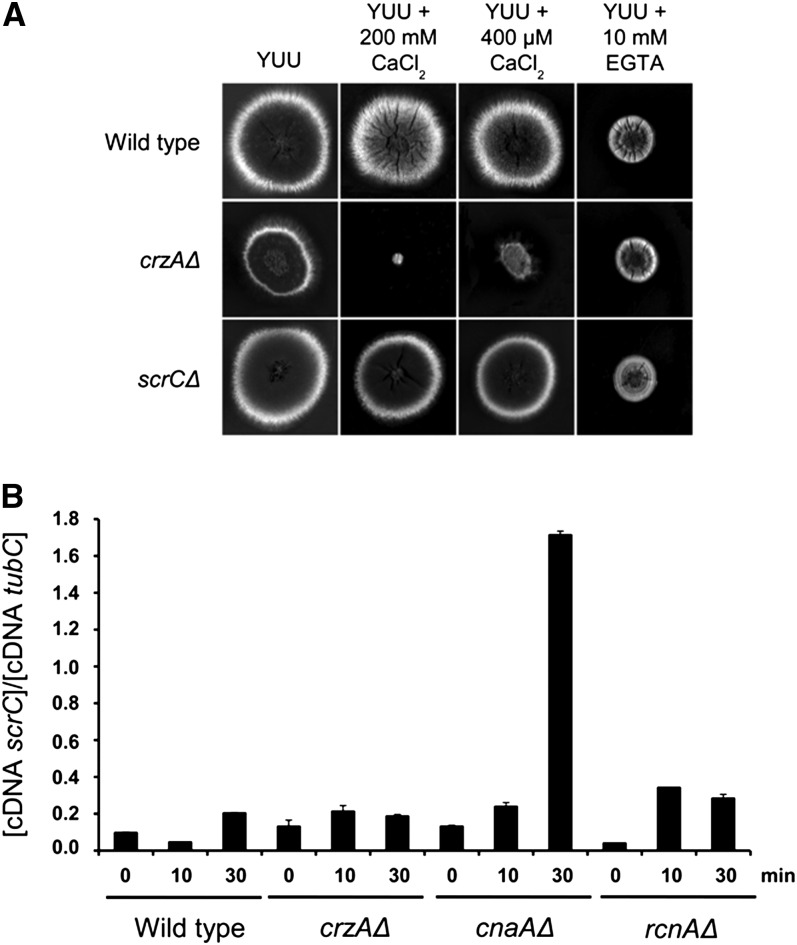
*scrC* mRNA levels and lack of detectable phenotype of *scrCΔ* single mutants. (A) Wild-type, *crzAΔ*, *cnaAΔ*, and *rcnAΔ* mutant strains were exposed to 200 mM CaCl_2_ for 0, 10 and 30 min; RNA extracted; and underwent reverse-transcription polymerase PCR for *scrC*. (B) Growth phenotypes for strains of the indicated relevant, partial genotypes on the indicated solid media after 72 hr at 30°.

The mutation now designated *scrC4* was located to the interval between *cbxA* (AN8830) and *halA* (AN8793) in linkage group III (File S1). Twenty-eight ORFs in this interval were sequenced in a *scrC4* strain and a G to A change was identified in the 3′ splice site of intron 4 of AN8823, encoding a hypothetical protein (Figure 7). This also revealed that the annotated AN8823 sequence was incorrect. cDNA and genomic sequencing plus homolog alignments indicated a 2571 nt coding region for a 857-residue protein with three 47 nt introns in addition to three large introns in the 5′ noncoding region. The corrected wild-type and mutant sequences are shown in Figure 7. The *scrC3* mutation is a −1 frameshift further downstream in the coding region (Figure 7). To confirm that these mutations in *scrC* were responsible for the suppression of *crzAΔ*, the coding region of *scrC* was deleted. *scrCΔ crzAΔ* strains were suppressed for calcium toxicity and pH 8 tolerance (Figure 1, A and C). This establishes that the *scrC* loss-of-function phenotype includes suppression of *crzAΔ* calcium toxicity and therefore shows that *scrC3* and -*4* are loss-of-function alleles. We have not observed any phenotypic differences between any of the *scrC*^-^ alleles and *scrC^+^* apart from *crzAΔ* suppression (Figures 4, 6C, and 8A and data not shown). A BlastP analysis of the ScrC sequence yielded ScrC homologs only in *Aspergilli* and other eurotiomycetes such as *Penicillium marfeneii*, *Paracoccidioides brasiliensis*, and *Histoplasma capsulatum* (Figure S3). We have been unable to detect similarity of ScrC to any protein of known function.

In an additional approach to provide information about *scrC* function, we investigated *scrC* mRNA levels when the *A. nidulans* wild-type, *cnaAΔ*, *crzAΔ*, and *rcnAΔ* mutant strains were exposed to 200 mM CaCl_2_ for 0, 10, and 30 min (Figure 8B). The *cnaA* and *rcnA* genes encode the calcineurin catalytic subunit and calcipressin (belonging to a class of endogenous calcineurin regulators), respectively (Soriani *et al.* 2008, 2010). There was only a modest (less than twofold) increase in *scrC* mRNA level when the wild-type or *crzAΔ* strains were exposed to calcium (Figure 8B). However, the *scrC* mRNA level increased 13-fold in the *cnaAΔ* strain and approximately threefold in the *rcnAΔ* strain upon calcium exposure (Figure 8B). In the absence of calcium exposure, the *scrC* mRNA level in the *rcnAΔ* strain was only about half that of the wild type. These results suggest that the modulation of the *scrC* mRNA accumulation upon calcium exposure is dependent upon calcineurin and, to a lesser extent, calcipressin.

### crzA overexpression does not suppress the cnaAΔ growth phenotype

**Figure 9:**
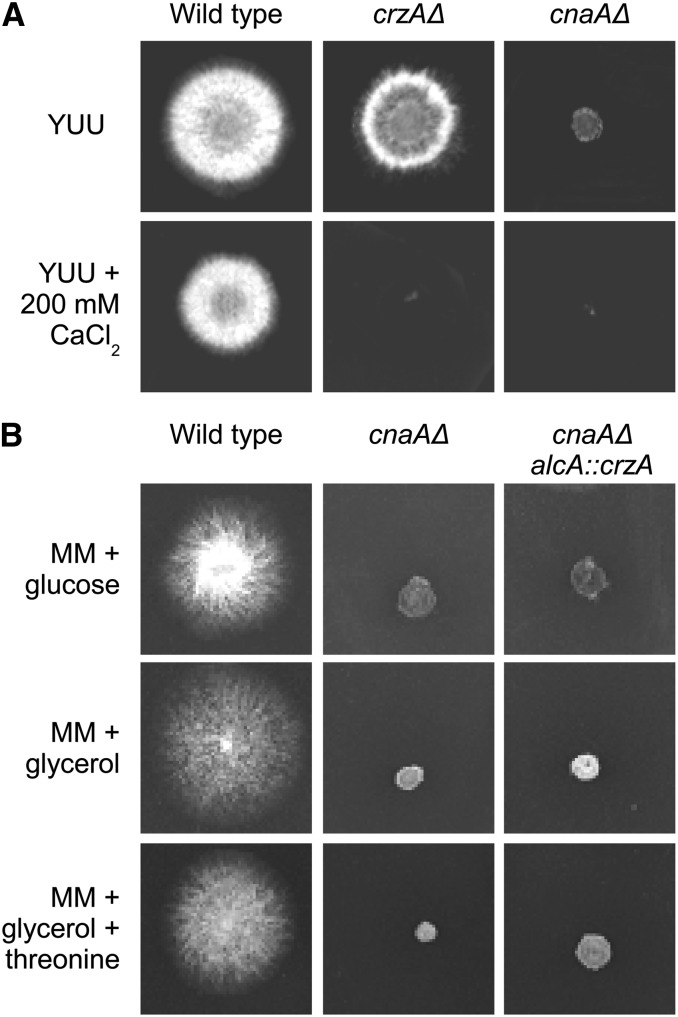
The *crzA* overexpression does not suppress the *cnaAΔ* growth phenotype. (A) Wild-type, *crzAΔ*, and *cnaAΔ* were grown either on YUU or YUU+200 mM CaCl_2_ for 72 hr at 30°. (B) Wild type, *cnaAΔ*, and *alcA*::*crzA cnaAΔ* were grown either on MM+glucose 4%, MM+glycerol 2%, or MM+glycerol 2%+threonine 100 mM for 72 hr at 30°.

To explore further the relationship between the regulatory domains of CrzA and calcineurin, we tested whether crzA overexpression was able to compensate for the lack of the calcineurin catalytic subunit. It has been shown that CnaA genetically interacts with CrzA and is responsible for the translocation of CrzA to the nucleus upon calcium stimulation in *A. nidulans* (Soriani *et al.* 2008, 2010; Spielvogel *et al.* 2008). The *cnaA*- and *crzA*-null mutants were both very sensitive to calcium toxicity yet show different phenotypes in the absence of exogenously added calcium, *i.e.*, *cnaAΔ* strains have a much smaller radial diameter than wild type and *ΔcrzA* strains (Figure 9A). The reduction in growth of the *cnaAΔ* mutant was attributed to increased hyphal branching and a reduction in polar expansion (Da Silva Ferreira *et al.* 2007; Soriani *et al.* 2008). To determine whether the *cnaAΔ* growth phenotype can be suppressed by excess CrzA, we overexpressed *crzA* using the *alcA* promoter in the *cnaAΔ* background (Figure 9B). The *alcA* promoter is repressed by glucose, derepressed by glycerol and induced to high levels by ethanol or L-threonine as sole carbon sources (Flipphi *et al.* 2002). *crzA* overexpression does not suppress the *cnaAΔ* reduced growth phenotype. This result suggests that in *A. nidulans* there are calcineurin targets that are independent from CrzA transcriptional activation.

## Discussion

We report the first attempt to use suppressor analyses to identify genes that interact genetically with Crz1/CrzA. A lack of CrzA confers considerable calcium sensitivity in *A. nidulans* (Soriani *et al.* 2008; Spielvogel *et al.* 2008), enabling the selection of suppressors by screening for calcium tolerance. In contrast to what is observed in *S. cerevisiae* (Stathopoulos and Cyert 1997), in *A. nidulans crzA* overexpression does not suppress the growth defect phenotype of a *cnaAΔ* strain (lacking the calcineurin catalytic subunit), suggesting the existence of *crzA*-independent functions controlled by calcineurin. The idea that there are CrzA/Crz1-independent functions controlled by calcineurin was first suggested in *C. albicans* (Bader *et al.* 2006; Karababa *Et Al*., 2006; Kullas *et al.*, 2007). Suppressors of *crzAΔ* calcium toxicity define at least three genes, *cnaB*, encoding the calcineurin regulatory subunit, *folA*, encoding dihydroneopterin reductase, and *scrC*, encoding a protein of unknown function. *cnaB1*, *folA1* and *scrCΔ* are not capable of suppressing the *cnaAΔ* mutant phenotype (data not shown).

The CnaA contains a globular catalytic domain, binding sites for both the regulatory calcineurin subunit B (CnaB) and calmodulin, in addition to an autoinhibitory region (Steinbach *et al.* 2007). The CnaA-CnaB heterodimer is maintained in an inactive state due to the binding of the C-terminal autoinhibitory domain of CNA to the catalytic cleft. Elevated cytosolic Ca^2+^ concentrations result in the concomitant binding of Ca^2+^ to CnaB and calmodulin, displacing the autoinhibitory domain, and thus activating the CnaA catalytic subunit (Steinbach *et al.* 2007). This investigation identified two mutant alleles of *cnaB* as suppressors of *crzAΔ*. Both mutational substitutions are located in EF-hand domains, suggesting that these changes affect the interaction between calcium and the regulatory subunit and possibly impact the activation of the catalytic subunit. Structural models of these modified proteins suggest that both substitutions can be tolerated without significant structural distortions. Both substitutions are possibly altering the interaction between catalytic and regulatory subunits of calcineurin. Either altered catalytic activity and/or modifications in the calcineurin substrate recognition might be involved in *crzAΔ* suppression. Docking interactions are very important for substrate and regulator recognition by calcineurin (Roy and Cyert 2009). Usually, either a small degenerate sequence or a conserved motif (PxIxIT) in the interacting protein directs binding to a docking site on the surface of the phosphatase distinct from the active site (Roy and Cyert 2009). These two kinds of modifications, increased flexibility for docking substrates and changed calcineurin activity might have an impact on the suppressor transcriptional landscape. In this context, it is worth noting that the mutated FolA shows some similarity with the small binding sequence for calcineurin (Figure S4). It is also possible to identify in FolA a secondary binding site, sharing some sequence similarity with those described for the interaction of NFAT with calcineurin (Hogan *et al.* 2003; Figure S4). The existence of several phosphorylatable serine- and threonine-containing motifs in FolA (Figure S2A) might lead to speculation that FolA itself might be a substrate for calcineurin.

The bases for suppression of *crzAΔ* calcium toxicity by the *folA1* mutation in the putative dihydroneopterin aldolase gene or by the loss-of-function mutations in *scrC* are obscure. The thermosensitive *folA1* mutation pleiotropically results in a mitotic blockage at restrictive temperature. It has been suggested that folate deficiency can accelerate telomere shortening, affect telomere function, and increase telomere-end fusions and subsequent breakage-fusion-bridge cycles (Fenech 2006; Moores *et al.* 2011). Chromatin and anaphase bridges are mitotic events that occur when telomeres of sister chromatids fuse together and subsequently fail to segregate completely into their respective daughter cells (Hoffelder *et al.* 2004; Chan and Hickson 2011). In higher eukaryotes, the DNA bridge connecting homologous chromosomes persists after the formation of individual daughter cells. When daughter cells exit mitosis and re-enter interphase, the chromatin bridge becomes known as an interphase bridge (Hoffelder *et al.* 2004; Chan and Hickson 2011). *A. nidulans*, like many lower eukaryotes, undergoes closed mitosis in which the nuclear envelope remains intact and mitosis occurs within the nucleus (De Souza and Osmani 2007). It is possible that what has been observed as a mitotic blockage is actually multiple occurrences of two nuclei kept together by a chromatin bridge within the nuclear envelope.

In summary, a plausible, but speculative, basis for how *cnaB1* and -*2* confer calcium tolerance to the *crzAΔ* strains are a modification in calcineurin activity, a change at the docking site or altered affinity for calcium binding. These modifications could affect transcriptional programs by activating one or more transcription factors able to control calcium homeostatic genes. In addition, it is also possible that direct dephosphorylation of proteins involved in signal transduction, such as kinases, could activate or repress signal cascades. Given that tetrahydrofolate is crucial to C1 metabolism, it is possible that the *folA1* mutation impacts histone methylation, which could affect gene expression. All these modifications could change calcium homeostasis by removing inhibitory concentrations of calcium from the cytoplasm through the action of putative calcium transporters that could either transport calcium to intracellular calcium stores or to the extracellular milieu. It is also possible that calcium uptake is decreased in these suppressor strains due to reduced expression of calcium channel encoding genes. The former possibility was documented, as *pmcA* and/or *pmcB* expression was increased in the *crzAΔ cnaB1*, *crzAΔ folA1*, and *crzAΔ scrC3* mutant strains postexposure to a short pulse of CaCl_2_. This is not observed for the *crzAΔ* single mutant, suggesting that decreased mRNA accumulation of these transporters is important for calcium sensitivity in this mutant. Finally, another possibility is that the extragenic suppression mutations are modifying posttranslational processes that affect calcium-tolerance, such as the protein stability of calcium channels. All these possibilities will be addressed in our laboratory to identify physiological mechanisms for observed suppression of the *crzA*-null mutation. However, the currently striking observation of this investigation is the importance of fungal calcineurin-dependent, CrzA-independent mechanisms of calcium tolerance.

## Supplementary Material

Supporting Information
