# Supplementary material for: Genetic Bypass of Aspergillus nidulans crzA Function in Calcium Homeostasis
Source: G3 (Bethesda). 2013 Jul 1;3(7):1129–41. doi: 10.1534/g3.113.005983 (PMC3704241; doi:10.1534/g3.113.005983)
Supplement: Supporting Information [file supp_3_7_1129__index.html]

Genetic Bypass of Aspergillus nidulans crzA Function in Calcium Homeostasis — Supporting Information 

# Genetic Bypass of *Aspergillus nidulans crzA* Function in Calcium Homeostasis

## Supporting Information for Almeida *et al.*, 2013

**Files in this Data Supplement:**

- Supporting Information - Figures S1-S4, File S1, and Tables S1-S2 (PDF, 1 MB)
- Figure S1 - Structural superposition of Human Calcineurin with the *A. nidulans* model (PDF, 626 KB)
- Figure S2 - Sequence conservation analysis of FolA (PDF, 650 KB)
- Figure S3 - Phylogenetic tree based on the amino acid alignment of AN8823 homologues (PDF, 162 KB)
- Figure S4 - (a) Calcineurin docking sequences in various interacting proteins (b) Sequence alignment of the calcineurin-binding region B in human NFAT1 to -4 and the calcineurin inhibitor DSCR1 (PDF, 189 KB)
- File S1 - Mapping of crzA suppressor mutations using classical genetic techniques (PDF, 139 KB)
- Table S1 - Primers and Lux probes used in this work (PDF, 99 KB)
- Table S2 - Analyses of the sexual crossings between the suppressors with wild-type strains (PDF, 129 KB)
