# Supplementary material for: Genetic Bypass of Aspergillus nidulans crzA Function in Calcium Homeostasis
Source: G3 (Bethesda). 2013 Jul 1;3(7):1129–41. doi: 10.1534/g3.113.005983 (PMC3704241; doi:10.1534/g3.113.005983)
Supplement: Supporting Information [file supp_g3.113.005983_005983SI.pdf]

G3 · Genes | Genomes | Genetics

Genetic bypass of *Aspergillus nidulans crzA* function in calcium homeostasis

Ricardo S. Almeida[†]*, Omar Loss**[†††], Ana Cristina Colabardini[†], Neil Andrew Brown[†], Elaine Bignell[†††], Marcela Savoldi[†], Sergio Pantano[††], Maria Helena S. Goldman[‡], Herbert N. Arst Jr[†††], and Gustavo H. Goldman[†]***

[†]Faculdade de Ciências Farmacêuticas de Ribeirão Preto, [‡]Faculdade de Filosofia, Ciências e Letras de Ribeirão Preto, Universidade de São Paulo, São Paulo, Brazil; [††]Biomolecular Simulations Group, Institut Pasteur de Montevideo, Uruguay; [†††]Section of Microbiology, Imperial College London, London SW7 2AZ, United Kingdom; ***Laboratório Nacional de Ciência e Tecnologia do Bioetanol – CTBE, Caixa Postal 6170, 13083-970 Campinas, São Paulo, Brazil

* Departamento de Microbiologia, Universidade Estadual de Londrina, Londrina, Brazil;
** Current address: Medical Research Council (MRC), Cell Biology Unit and Laboratory for Molecular and Cell Biology, Department of Cell and Developmental Biology, University College London, London WC1E 6BT, UK

Corresponding author: Dr. Gustavo H. Goldman
Departamento de Ciências Farmacêuticas
Faculdade de Ciências Farmacêuticas de Ribeirão Preto
Universidade de São Paulo
Av. do Café S/N
CEP 14040-903, Ribeirão Preto, São Paulo, Brazil
Phone: 0055-016-36024280
Fax: 0055-016-36024280
e-mail address: ggoldman@usp.br

DOI: 10.1534/g3.113.005983

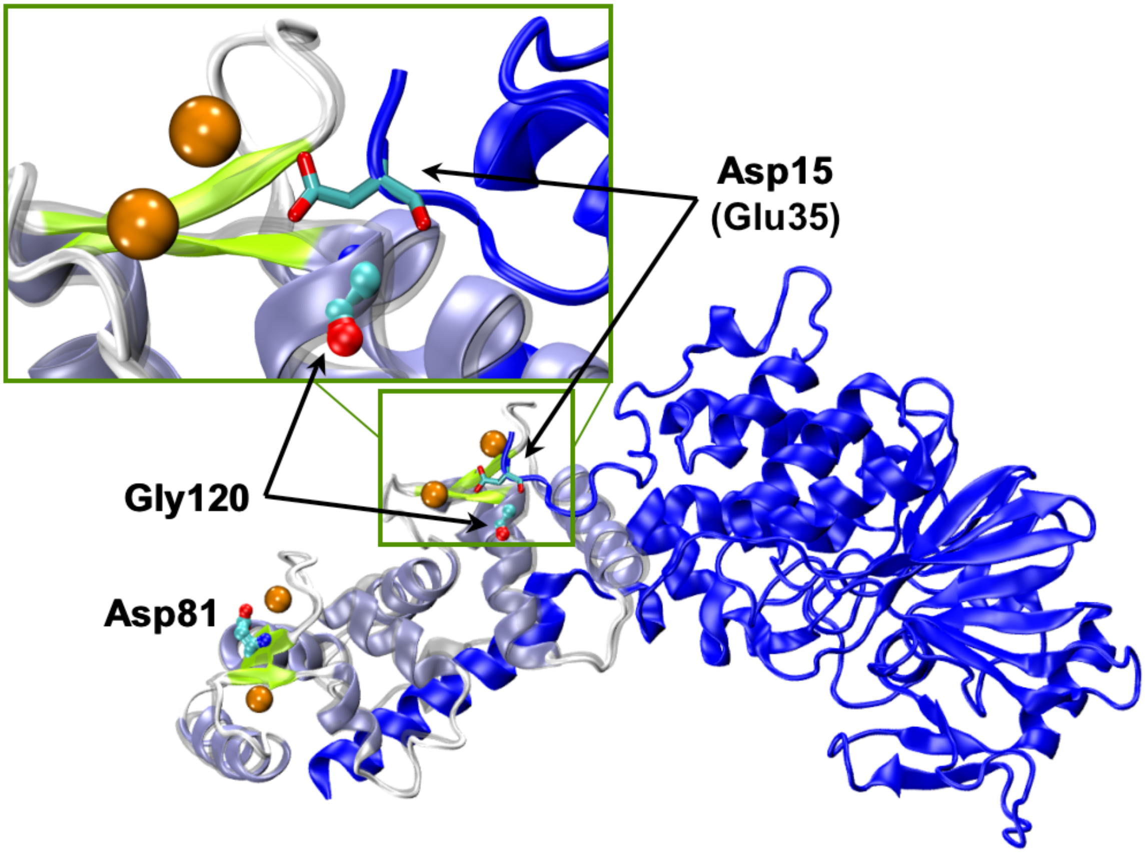

Figure S1 Structural superposition of Human Calcineurin with the *A. nidulans* model. The comparative model of the regulatory subunit of *A. nidulans* calcineurin is presented with the same colouring scheme as Figure 5 (main text). Residues Asp81 and Gly120 are shown in balls and sticks. The structure of the regulatory subunit of the human homologue is superimposed and shown as grey, semi-transparent cartoon. The catalytic subunit is shown in blue. The helix attached to the regulatory subunit corresponds to the autoinhibitory segment. The acidic residue (Asp15 in the human protein, corresponding to Glu35 in A. *nidulans*), which is in the close neighborhood of Gly12 is shown in sticks. The inset shows a closer view of this interaction in a slightly different orientation.

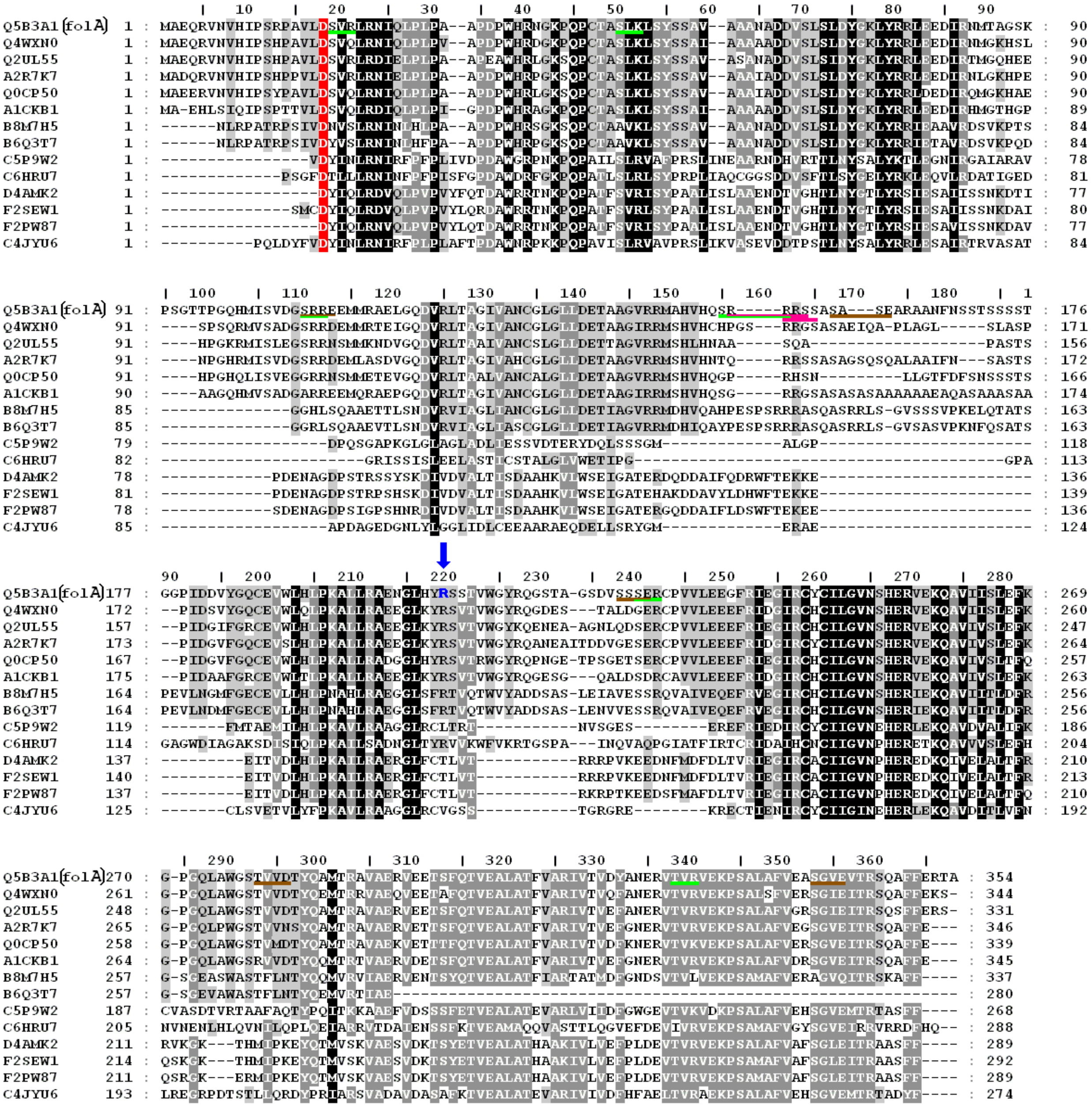

Figure S2A Sequence conservation analysis of FolA.

A PSI-BLAST search was made using the primary sequence of FolA on the UniProt database (www.uniprot.org). Sequences were selected up to E-value = 0.0001. The list of aligned sequences, coloured by conservation, are shown below. Black, dark grey, light grey and white background shading indicate decreasing degrees of conservation. The conserved Asp18, which is mutationally substituted by Asn is indicated by a red background. Underlined residues are sequence motifs which are potentially phosphorylatable by PKA (pink), PKC (green) and casein kinase II (brown).

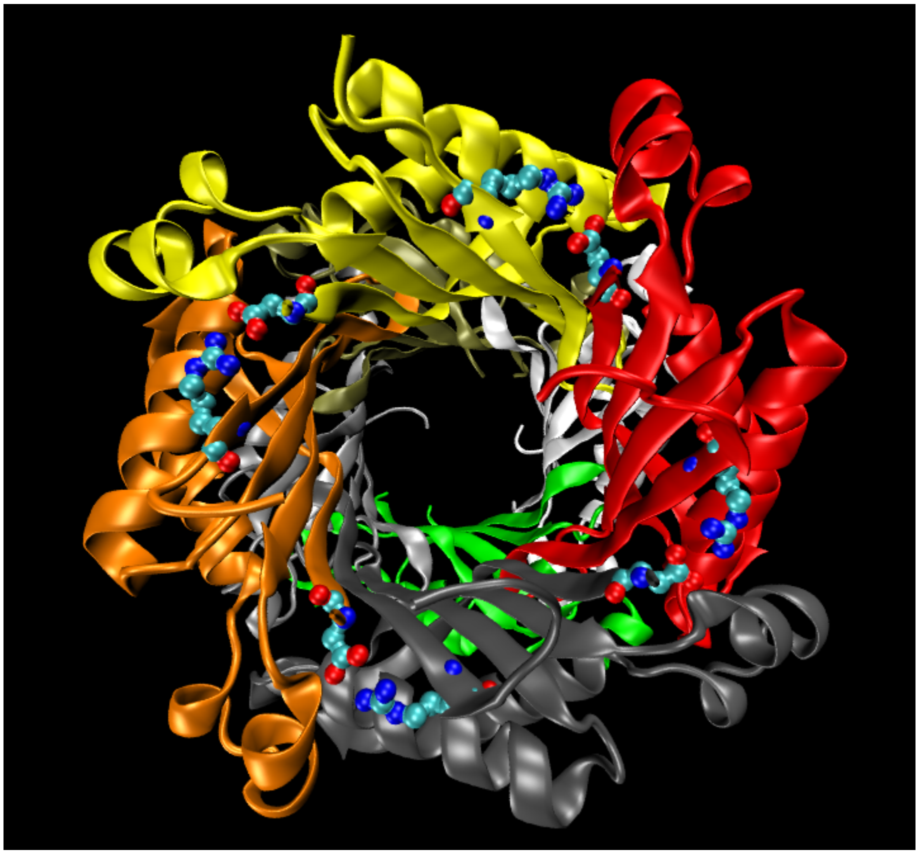

Figure S2B The low level of identity between FolA and experimentally characterised protein homologues makes the construction of theoretical models difficult. However, comparison of the position occupied by Asp18 with analogous residues in different homologues reveals that this conserved aspartic acid establishes an intermolecular salt bridge with a conserved arginine (Arg207 in FolA) located in a neighbouring subunit (see below). This arginine is indicated by a blue arrow in the multiple sequence alignment. Although it is unlikely that the rather conservative D18N mutation alters the protein structure significantly, it might play a role in the formation of its functional oligomeric form. The figure shows the crystal structure of 7,8-dihydroneopterin aldolase from *Staphylococcus aureus* (PDB id:1DHN, **Crystal structure and reaction mechanism of 7,8-dihydroneopterin aldolase from *Staphylococcus aureus.* Hennig, M., D'Arcy, A., Hampele, I.C., Page, M.G., Oefner, C., Dale, G.E. Nat. Struct. Biol. (1998) 5: 357-362**). Each of the eight protein monomers is represented by a different colour. Asp3 and Arg118 (corresponding to Asp18 and Arg207 in FolA, respectively) are shown using balls and sticks.

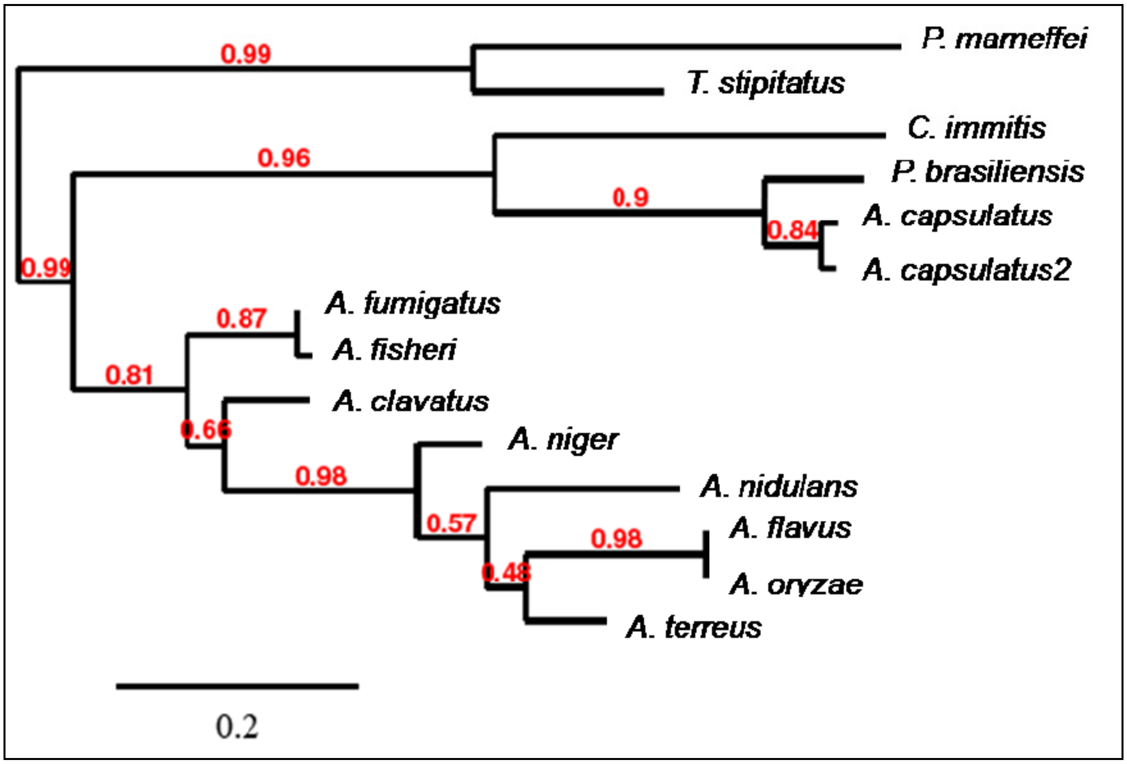

Figure S3 Phylogenetic tree based on the amino acid alignment of AN8823 homologues. Phylogenetic tree calculated using the Bootseq, Protdist and Neighbour programs in the Phylip package. The bootstrap values calculated are shown beside their respective branches.

a)

**Consensus**	PxIxIT
VIVIT	PVIVIT
NFAT1	PRIEIT
TRESK	PQIIIS
Crz1	PIISIQ
Slm1	PNIYIQ
Slm2	PEFYIE
Hph1	PVIAVN
RCAN1	PSVVVH
Rcn1	GAITID
Rcn2	PSITVN
Rcn1 (Dm)	PAIIVH
**folA (mut)**	PAVLNS

b)

NFAT1	ESILLVPP-----TW--PKPLVP
NFAT2	DQYLAVPQ--HPYQWAKPKPLSP
NFAT3	MDYLAVPS---PLAWSKARIGGH
NFAT4	DQFLSVPS---PFTWSKPKPGHT
DSCR1	KQFLISPPASPPVGWKQVEDATP
**folA**	LRNIQLPLPAAPDPWHRNGKPQP

Figure S4 a) Calcineurin docking sequences in various interacting proteins (adapted from Interaction of calcineurin with substrates and targeting proteins. Huiming Li, Anjana Rao and Patrick G. Hogan, Trends Cell Biol., 2011, 21: 91-103). The mutated residue encoded by *folA1* (D18N) is highlighted in red. b) Sequence alignment of the calcineurin-binding region B in human NFAT1 to -4 and the calcineurin inhibitor DSCR1 (taken from Transcriptional regulation by calcium, calcineurin, and NFAT. Patrick G. Hogan, Lin Chen, Julie Nardone, et al. *Genes Dev.* 2003, 17: 2205-2232). These sequences were realigned including the sequence of FolA using T-coffee with standard options at (http://www.tcoffee.org/ XXREF T-Coffee: a web server for the multiple sequence alignment of protein and RNA sequences using structural information and homology extension. Paolo Di Tommaso, Sebastien Moretti, Ioannis Xenarios, Miquel Orobitg, Alberto Montanyola, Jia-Ming Chang, Jean-François Taly and Cedric Notredame, Nucl. Acids Res. 2011, 39 (suppl 2): W13-W17).

File S1

Mapping of crzA suppressor mutations using classical genetic techniques

The standard parasexual and sexual cycle mapping techniques (Clutterbuck, 1974) were used in conjunction with Clutterbuck's genetic map (www.fgsc.net/Aspergillus/gene_list/index.html) and the (partially) annotated genome sequence (www.aspgd.org/) to determine map positions relative to those of identified genes.

Genetic mapping of *cna*B: The thermosensitive mutation provisionally designated *scr*A1 and now designated *cna*B1 and the mutation provisionally designated rev2 and now designated *cna*B2 were both located to linkage group I using the parasexual cycle. As both of these mutations showed close linkage to *paba*A1 in preliminary crosses and might therefore be allelic, further localization was done only with *cna*B1. Using selected progeny a cross of genotype *bi*A1 *lys*F51 x *y*A2 *cna*B2 *paba*A1 was analysed. Of 51 *paba*A$^+$ *lysF*$^+$ progeny, 3 carried *cna*B2, 9 carried *y*A2 and 40 carried *bi*A1; of 51 *cna*B$^+$ *lys*F$^+$ progeny, 24 carried *paba*A1, 15 carried *y*A2 and 36 carried *bi*A1; of 12 *cna*B+ *paba*A$^+$ progeny, 11 carried *lys*F51, 11 carried *y*A2 and 3 carried *bi*A1. These data clearly indicate the order *bi*A—*y*A—*cna*B—*paba*A—*lys*F.

Genetic mapping of *fol*A: The thermosensitive mutation provisionally designated *scr*B2 and now designated *fol*A1 was located to linkage group III using the parasexual cycle. As *scr*C is also in linkage group III (see below), a *fol*A1 x *scr*C3 cross was analysed and indicated free recombination as did a *fol*A1 cross to a strain carrying the *scr*C-linked *hal*A24 and *cbx*A17 mutations. Following crosses indicating free recombination between *fol*A1 and a number of other linkage group III markers, random progeny from a cross involving *mea*B6, *cnx*H3 and *s*C12 suggested linkage and the order *fol*A—*mea*B—*cnx*H—*s*C. A cross involving *fol*A1 and *gal*E9, *mea*B6, *cnx*H3 and *s*C12 gave inconclusive results with regard to gene order with a slight and misleading (owing to insufficient numbers of progeny) indication that *fol*A might lie between *gal*E and *mea*B. Analysis of progeny able to grow at 42° C from a cross of partial genotype *gal*E9 *fol*A1 *mea*B6 *s*C12 x *nud*R825 established that *fol*A is centromere-distal to *nud*R. Finally, analysis of 56 *gap*A$^+$ *fol*A$^+$ progeny from a cross of relevant partial genotype *gap*AΔ x *fol*A1 *gal*E9 *mea*B6 *cnx*H3 *s*C12 showed that 4 carried *gal*E9, 9 carried *mea*B6 and 11 carried *cnx*H3 and along with analysis of 100 random progeny showed that the *gap*A to *gal*E map distance is greater than either the *gap*A to *fol*A or the *fol*A to *gal*E map distance. Taken together these mapping crosses plus the genome sequence indicate the gene order as *gap*A—*fol*A—*gal*E—*nud*R—*mea*B—*cnx*H—*s*C.

Genetic mapping of *scr*C: Mutations provisionally designated rev1 and rev3 and now designated *scr*C4 and *scr*C3, respectively, were located to linkage group III using the parasexual cycle. As these suppressor mutations have similar phenotypes, meiotic analysis was done only with *scrC*3. The first cross to be analysed established close linkage between scrC3 and *cbx*A17. A subsequent cross involving *scr*C3, *cbx*A17 and *hal*AΔ established that *scr*C lies between *cbx*A and *hal*A in close proximity to *hal*A.

Table S1 Primers and Lux probes used in this work

An8823 pRS426 5F	5’ GTAACGCCAGGGTTTTCCCAGTCACGACGGTTCACAGGTGGATGGAGC 3’
An8823 pyro 5R	5’ GACCCAACAACCATGATACCACTCGGCCACTTATCACTCAAC 3’
An8823 pyro 3F	5’ CTGTCGATCATGTGGATGCTGTTGGCTATCAGAATTCTGGGTTTAG 3’
An8823 pRS426 3R	5’ GCGGATAACAATTTCACACAGGAAACAGCCTATCCTTATTCGCAACTCCCTGC 3’
An8823_RL	5´ GAGATACGAGACGCAGGTCCG[FAM]G 3´
An8823_FL/RL	5´ CCCGTGGCTTCCAACAACAT 3´
An8823 pRS 5F	5’ GTAACGCCAGGGTTTTCCCAGTCACGACGGTTCACAGGTGGATGGAGC 3’
An8823 pyro 5R	5’ GACCCAACAACCATGATACCACTCGGCCACTTATCACTCAAC 3’
An8823 pyro 3F	5’ CTGTCGATCATGTGGATGCTGTTGGCTATCAGAATTCTGGGTTTAG 3’
An8823 pRS 3R	5’ GCGGATAACAATTTCACACAGGAAACAGCCTATCCTTATTCGCAACTCCCTGC 3’
pmcA probe Lux	5’-CGGACCTTCAATGCCTGGTTGTC[FAM]G 3´
pmcA primer Lux	5’-GGGAGGCGTTCAAGTTCGAT-3’
pmcB probe Lux	5’-CGGATTCTTCCAACCCAGACATC[FAM]G 3´
pmcB primer Lux	5’-CCAGACAAAGGTGTTGAAGACGA-3’

Table S2 Analyses of the sexual crossings between the suppressors with wild-type strains

Parentals		Phenotypes				Total
		$Ca^R ts^+$	$Ca^R ts^-$	$Ca^S ts^+$	$Ca^S ts^-$	
folA1 crzAΔ	GR5	108	131	53	0	292
cnaB1 crzAΔ	R21	255	22	19	0	296

$Ca^R ts^+$, calcium-resistant and thermoresistant to 44°C. $Ca^R ts^-$, calcium-resistant and thermosensitive to 44°C. $Ca^S ts^+$, calcium-sensitive and thermoresistant to 44°C. $Ca^S ts^-$, calcium-sensitive and thermosensitive to 44°C.
