# Supplementary material for: Genetic Bypass of Aspergillus nidulans crzA Function in Calcium Homeostasis
Source: G3 (Bethesda). 2013 Jul 1;3(7):1129–41. doi: 10.1534/g3.113.005983 (PMC3704241; doi:10.1534/g3.113.005983)
Supplement: Supporting Information [file supp_g3.113.005983_FigureS1.pdf]

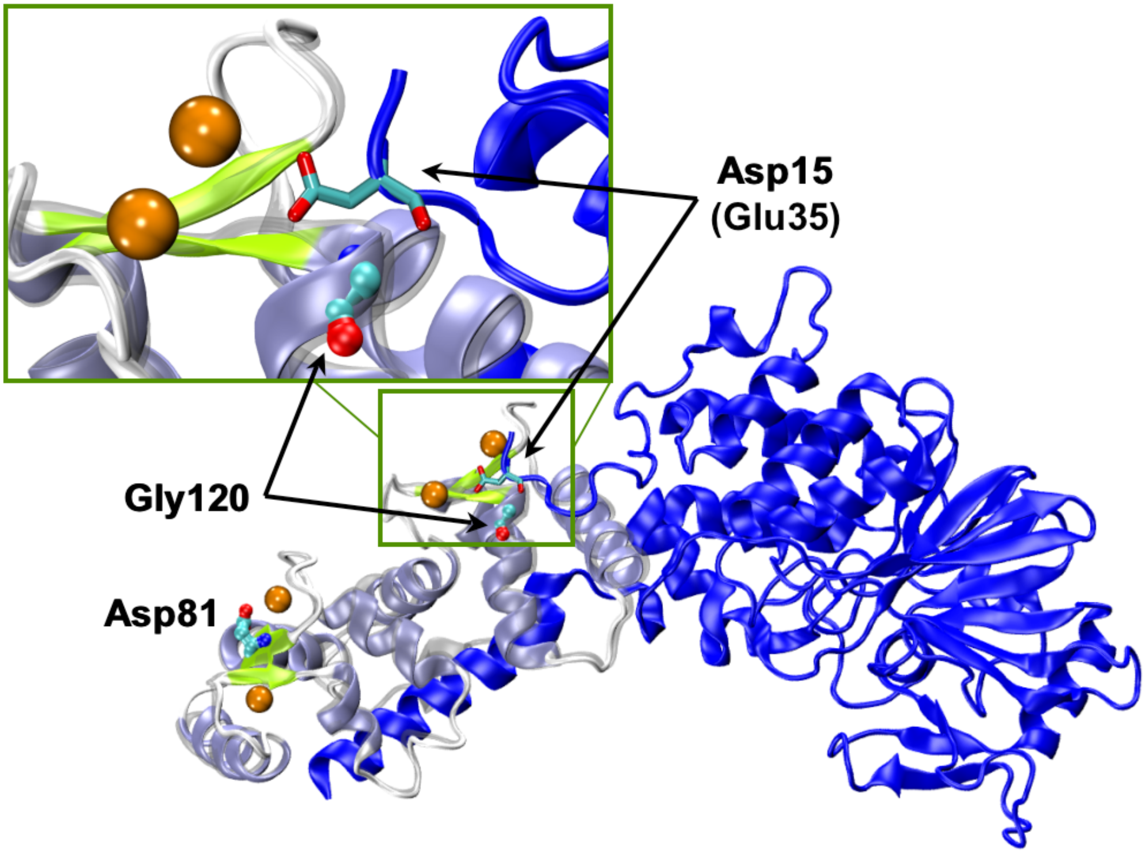

Figure S1 Structural superposition of Human Calcineurin with the *A. nidulans* model. The comparative model of the regulatory subunit of *A. nidulans* calcineurin is presented with the same colouring scheme as Figure 5 (main text). Residues Asp81 and Gly120 are shown in balls and sticks. The structure of the regulatory subunit of the human homologue is superimposed and shown as grey, semi-transparent cartoon. The catalytic subunit is shown in blue. The helix attached to the regulatory subunit corresponds to the autoinhibitory segment. The acidic residue (Asp15 in the human protein, corresponding to Glu35 in A. *nidulans*), which is in the close neighborhood of Gly12 is shown in sticks. The inset shows a closer view of this interaction in a slightly different orientation.
