# Supplementary material for: Genetic Bypass of Aspergillus nidulans crzA Function in Calcium Homeostasis
Source: G3 (Bethesda). 2013 Jul 1;3(7):1129–41. doi: 10.1534/g3.113.005983 (PMC3704241; doi:10.1534/g3.113.005983)
Supplement: Supporting Information [file supp_g3.113.005983_FigureS2.pdf]

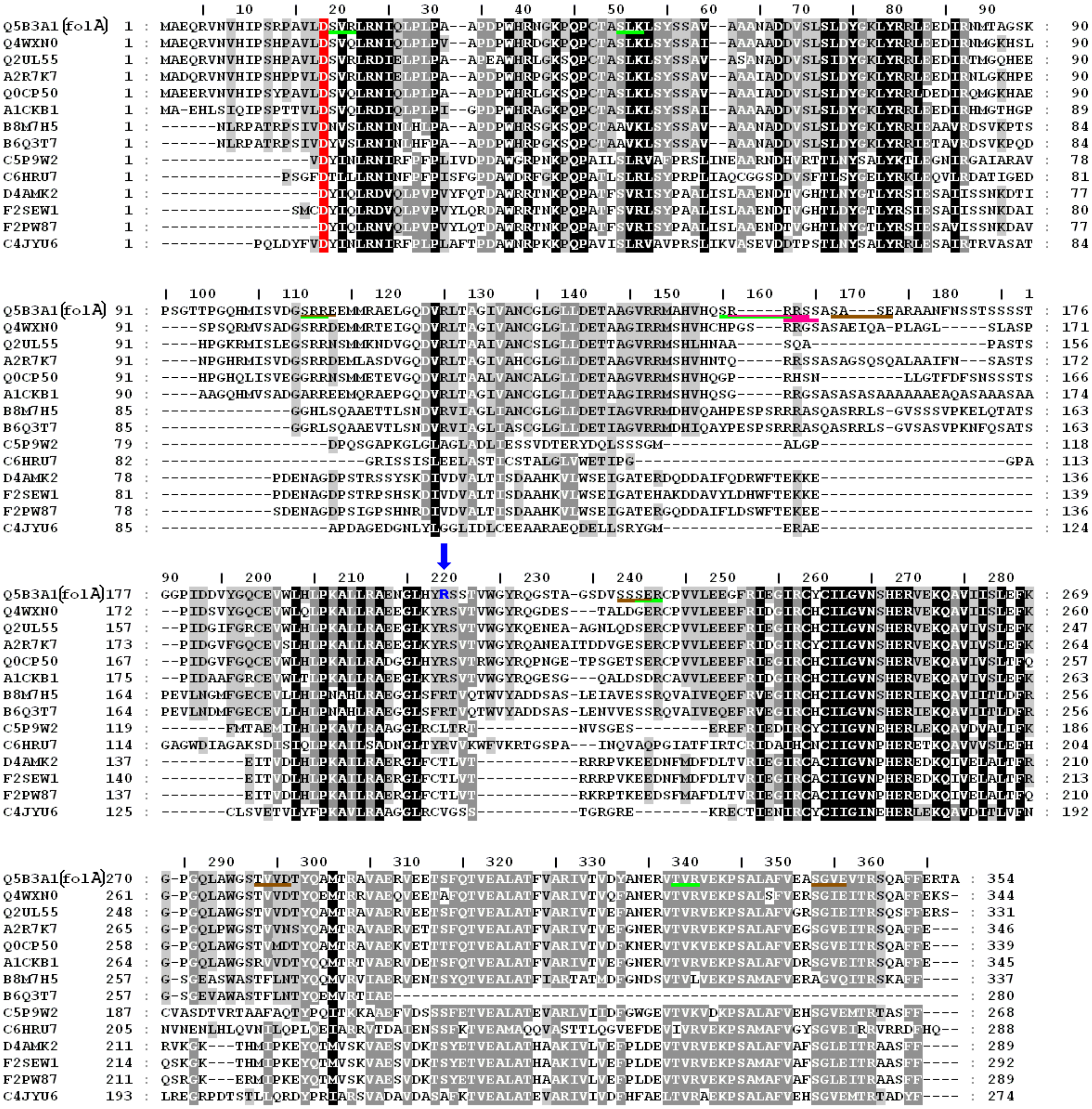

Figure S2A Sequence conservation analysis of FolA.
A PSI-BLAST search was made using the primary sequence of FolA on the UniProt database (www.uniprot.org). Sequences were selected up to E-value = 0.0001. The list of aligned sequences, coloured by conservation, are shown below. Black, dark grey, light grey and white background shading indicate decreasing degrees of conservation. The conserved Asp18, which is mutationally substituted by Asn is indicated by a red background. Underlined residues are sequence motifs which are potentially phosphorylatable by PKA (pink), PKC (green) and casein kinase II (brown).

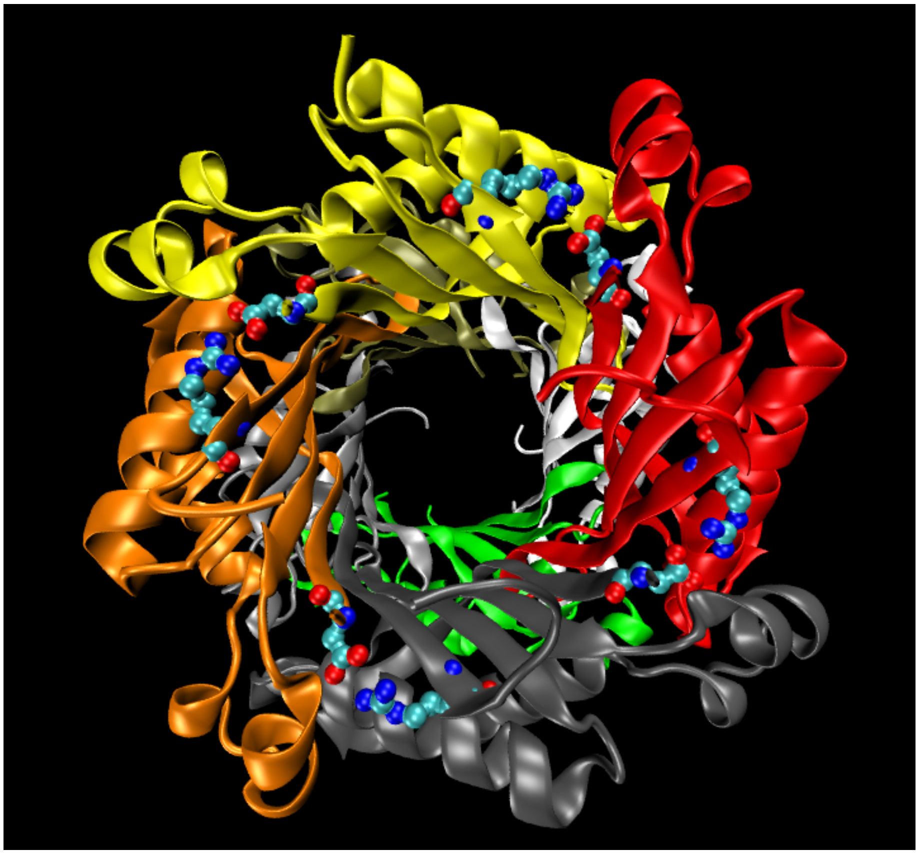

Figure S2B The low level of identity between FolA and experimentally characterised protein homologues makes the construction of theoretical models difficult. However, comparison of the position occupied by Asp18 with analogous residues in different homologues reveals that this conserved aspartic acid establishes an intermolecular salt bridge with a conserved arginine (Arg207 in FolA) located in a neighbouring subunit (see below). This arginine is indicated by a blue arrow in the multiple sequence alignment. Although it is unlikely that the rather conservative D18N mutation alters the protein structure significantly, it might play a role in the formation of its functional oligomeric form. The figure shows the crystal structure of 7,8-dihydroneopterin aldolase from *Staphylococcus aureus* (PDB id:1DHN, **Crystal structure and reaction mechanism of 7,8-dihydroneopterin aldolase from *Staphylococcus aureus.* Hennig, M., D'Arcy, A., Hampele, I.C., Page, M.G., Oefner, C., Dale, G.E. Nat. Struct. Biol. (1998) 5: 357-362**). Each of the eight protein monomers is represented by a different colour. Asp3 and Arg118 (corresponding to Asp18 and Arg207 in FolA, respectively) are shown using balls and sticks.
