# Supplementary material for: Genetic Bypass of Aspergillus nidulans crzA Function in Calcium Homeostasis
Source: G3 (Bethesda). 2013 Jul 1;3(7):1129–41. doi: 10.1534/g3.113.005983 (PMC3704241; doi:10.1534/g3.113.005983)
Supplement: Supporting Information [file supp_g3.113.005983_FigureS3.pdf]

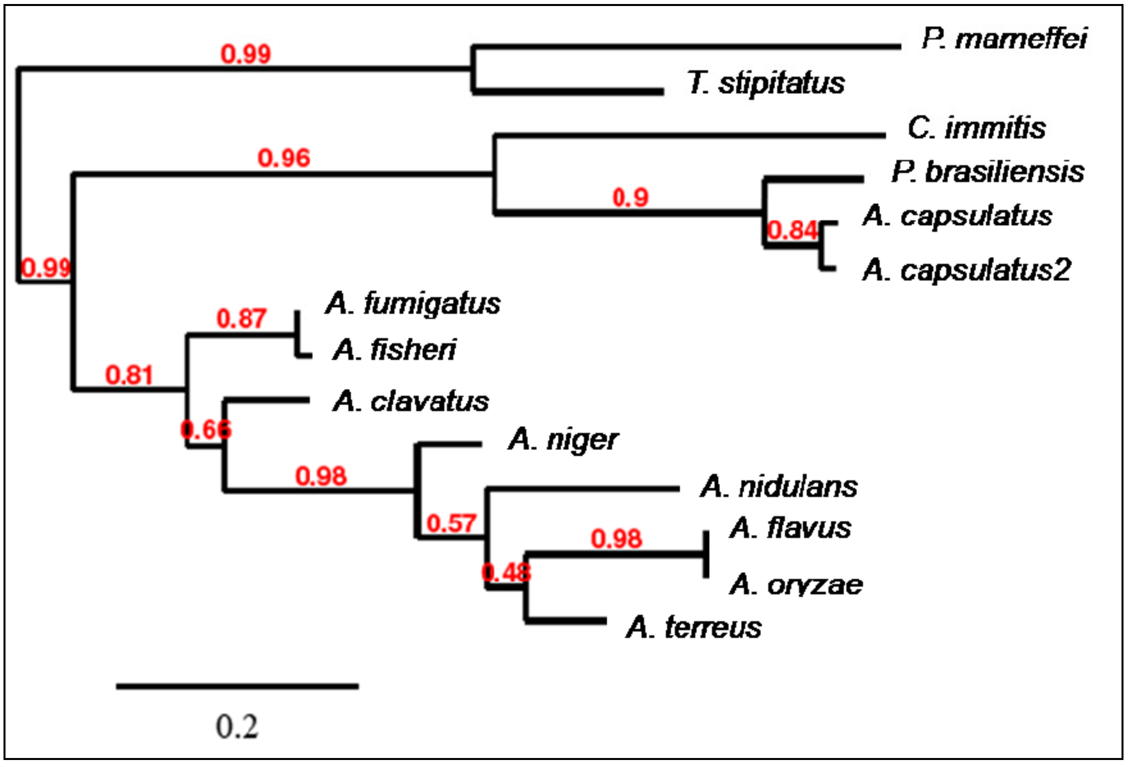

Figure S3 Phylogenetic tree based on the amino acid alignment of AN8823 homologues. Phylogenetic tree calculated using the Bootseq, Protdist and Neighbour programs in the Phylip package. The bootstrap values calculated are shown beside their respective branches.
