# Supplementary material for: Genetic Bypass of Aspergillus nidulans crzA Function in Calcium Homeostasis
Source: G3 (Bethesda). 2013 Jul 1;3(7):1129–41. doi: 10.1534/g3.113.005983 (PMC3704241; doi:10.1534/g3.113.005983)
Supplement: Supporting Information [file supp_g3.113.005983_FigureS4.pdf]

a)

**Consensus**	PxIxIT
VIVIT	PVIVIT
NFAT1	PRIEIT
TRESK	PQIIIS
Crz1	PIISIQ
Slm1	PNIYIQ
Slm2	PEFYIE
Hph1	PVIAVN
RCAN1	PSVVVH
Rcn1	GAITID
Rcn2	PSITVN
Rcn1 (Dm)	PAIIVH
**folA (mut)**	PAVLNS

b)

NFAT1	ESILLVPP-----TW--PKPLVP
NFAT2	DQYLAVPQ--HPYQWAKPKPLSP
NFAT3	MDYLAVPS---PLAWSKARIGGH
NFAT4	DQFLSVPS---PFTWSKPKPGHT
DSCR1	KQFLISPPASPPVGWKQVEDATP
**folA**	LRNIQLPLPAAPDPWHRNGKPQP

Figure S4 a) Calcineurin docking sequences in various interacting proteins (adapted from Interaction of calcineurin with substrates and targeting proteins. Huiming Li, Anjana Rao and Patrick G. Hogan, Trends Cell Biol., 2011, 21: 91-103). The mutated residue encoded by *folA1* (D18N) is highlighted in red. b) Sequence alignment of the calcineurin-binding region B in human NFAT1 to -4 and the calcineurin inhibitor DSCR1 (taken from Transcriptional regulation by calcium, calcineurin, and NFAT. Patrick G. Hogan, Lin Chen, Julie Nardone, et al. *Genes Dev.* 2003, 17: 2205-2232). These sequences were realigned including the sequence of FolA using T-coffee with standard options at (http://www.tcoffee.org/ XXREF T-Coffee: a web server for the multiple sequence alignment of protein and RNA sequences using structural information and homology extension. Paolo Di Tommaso, Sebastien Moretti, Ioannis Xenarios, Miquel Orobitg, Alberto Montanyola, Jia-Ming Chang, Jean-François Taly and Cedric Notredame, Nucl. Acids Res. 2011, 39 (suppl 2): W13-W17).
