# Supplementary material for: Genetic Bypass of Aspergillus nidulans crzA Function in Calcium Homeostasis
Source: G3 (Bethesda). 2013 Jul 1;3(7):1129–41. doi: 10.1534/g3.113.005983 (PMC3704241; doi:10.1534/g3.113.005983)
Supplement: Supporting Information [file supp_g3.113.005983_FileS1.pdf]

File S1

Mapping of crzA suppressor mutations using classical genetic techniques

The standard parasexual and sexual cycle mapping techniques (Clutterbuck, 1974) were used in conjunction with Clutterbuck's genetic map (www.fgsc.net/Aspergillus/gene_list/index.html) and the (partially) annotated genome sequence (www.aspgd.org/) to determine map positions relative to those of identified genes.

Genetic mapping of *cna*B: The thermosensitive mutation provisionally designated *scr*A1 and now designated *cna*B1 and the mutation provisionally designated rev2 and now designated *cna*B2 were both located to linkage group I using the parasexual cycle. As both of these mutations showed close linkage to *paba*A1 in preliminary crosses and might therefore be allelic, further localization was done only with *cna*B1. Using selected progeny a cross of genotype *bi*A1 *lys*F51 x *y*A2 *cna*B2 *paba*A1 was analysed. Of 51 *paba*A$^+$ *lysF*$^+$ progeny, 3 carried *cna*B2, 9 carried *y*A2 and 40 carried *bi*A1; of 51 *cna*B$^+$ *lys*F$^+$ progeny, 24 carried *paba*A1, 15 carried *y*A2 and 36 carried *bi*A1; of 12 *cna*B+ *paba*A$^+$ progeny, 11 carried *lys*F51, 11 carried *y*A2 and 3 carried *bi*A1. These data clearly indicate the order *bi*A—*y*A—*cna*B—*paba*A—*lys*F.

Genetic mapping of *fol*A: The thermosensitive mutation provisionally designated *scr*B2 and now designated *fol*A1 was located to linkage group III using the parasexual cycle. As *scr*C is also in linkage group III (see below), a *fol*A1 x *scr*C3 cross was analysed and indicated free recombination as did a *fol*A1 cross to a strain carrying the *scr*C-linked *hal*A24 and *cbx*A17 mutations. Following crosses indicating free recombination between *fol*A1 and a number of other linkage group III markers, random progeny from a cross involving *mea*B6, *cnx*H3 and *s*C12 suggested linkage and the order *fol*A—*mea*B—*cnx*H—*s*C. A cross involving *fol*A1 and *gal*E9, *mea*B6, *cnx*H3 and *s*C12 gave inconclusive results with regard to gene order with a slight and misleading (owing to insufficient numbers of progeny) indication that *fol*A might lie between *gal*E and *mea*B. Analysis of progeny able to grow at 42° C from a cross of partial genotype *gal*E9 *fol*A1 *mea*B6 *s*C12 x *nud*R825 established that *fol*A is centromere-distal to *nud*R. Finally, analysis of 56 *gap*A$^+$ *fol*A$^+$ progeny from a cross of relevant partial genotype *gap*AΔ x *fol*A1 *gal*E9 *mea*B6 *cnx*H3 *s*C12 showed that 4 carried *gal*E9, 9 carried *mea*B6 and 11 carried *cnx*H3 and along with analysis of 100 random progeny showed that the *gap*A to *gal*E map distance is greater than either the *gap*A to *fol*A or the *fol*A to *gal*E map distance. Taken together these mapping crosses plus the genome sequence indicate the gene order as *gap*A—*fol*A—*gal*E—*nud*R—*mea*B—*cnx*H—*s*C.

Genetic mapping of *scr*C: Mutations provisionally designated rev1 and rev3 and now designated *scr*C4 and *scr*C3, respectively, were located to linkage group III using the parasexual cycle. As these suppressor mutations have similar phenotypes, meiotic analysis was done only with *scrC*3. The first cross to be analysed established close linkage between scrC3 and *cbx*A17. A subsequent cross involving *scr*C3, *cbx*A17 and *hal*AΔ established that *scr*C lies between *cbx*A and *hal*A in close proximity to *hal*A.
