# Supplementary material for: Genetic Bypass of Aspergillus nidulans crzA Function in Calcium Homeostasis
Source: G3 (Bethesda). 2013 Jul 1;3(7):1129–41. doi: 10.1534/g3.113.005983 (PMC3704241; doi:10.1534/g3.113.005983)
Supplement: Supporting Information [file supp_g3.113.005983_TableS1.pdf]

Table S1 Primers and Lux probes used in this work

An8823 pRS426 5F	5’ GTAACGCCAGGGTTTTCCCAGTCACGACGGTTCACAGGTGGATGGAGC 3’
An8823 pyro 5R	5’ GACCCAACAACCATGATACCACTCGGCCACTTATCACTCAAC 3’
An8823 pyro 3F	5’ CTGTCGATCATGTGGATGCTGTTGGCTATCAGAATTCTGGGTTTAG 3’
An8823 pRS426 3R	5’ GCGGATAACAATTTCACACAGGAAACAGCCTATCCTTATTCGCAACTCCCTGC 3’
An8823_RL	5´ GAGATACGAGACGCAGGTCCG[FAM]G 3´
An8823_FL/RL	5´ CCCGTGGCTTCCAACAACAT 3´
An8823 pRS 5F	5’ GTAACGCCAGGGTTTTCCCAGTCACGACGGTTCACAGGTGGATGGAGC 3’
An8823 pyro 5R	5’ GACCCAACAACCATGATACCACTCGGCCACTTATCACTCAAC 3’
An8823 pyro 3F	5’ CTGTCGATCATGTGGATGCTGTTGGCTATCAGAATTCTGGGTTTAG 3’
An8823 pRS 3R	5’ GCGGATAACAATTTCACACAGGAAACAGCCTATCCTTATTCGCAACTCCCTGC 3’
pmcA probe Lux	5’-CGGACCTTCAATGCCTGGTTGTC[FAM]G 3´
pmcA primer Lux	5’-GGGAGGCGTTCAAGTTCGAT-3’
pmcB probe Lux	5’-CGGATTCTTCCAACCCAGACATC[FAM]G 3´
pmcB primer Lux	5’-CCAGACAAAGGTGTTGAAGACGA-3’
