# Supplementary material for: Genetic Bypass of Aspergillus nidulans crzA Function in Calcium Homeostasis
Source: G3 (Bethesda). 2013 Jul 1;3(7):1129–41. doi: 10.1534/g3.113.005983 (PMC3704241; doi:10.1534/g3.113.005983)
Supplement: Supporting Information [file supp_g3.113.005983_TableS2.pdf]

Table S2 Analyses of the sexual crossings between the suppressors with wild-type strains

Parentals		Phenotypes				Total
		$Ca^R ts^+$	$Ca^R ts^-$	$Ca^S ts^+$	$Ca^S ts^-$	
folA1 crzAΔ	GR5	108	131	53	0	292
cnaB1 crzAΔ	R21	255	22	19	0	296

$Ca^R ts^+$, calcium-resistant and thermoresistant to 44°C. $Ca^R ts^-$, calcium-resistant and thermosensitive to 44°C. $Ca^S ts^+$, calcium-sensitive and thermoresistant to 44°C. $Ca^S ts^-$, calcium-sensitive and thermosensitive to 44°C.
